# DaiTongXiao improves gout nephropathy by inhibiting inflammatory response through the TLR4/MyD88/NF-κB pathway

**DOI:** 10.3389/fphar.2024.1447241

**Published:** 2024-08-07

**Authors:** Feifan Liu, Yuanmei Bai, Yan Wan, Shifang Luo, Linao Zhang, Xue Wu, Rong Chen, Zili Yin, Yuhuan Xie, Peixin Guo

**Affiliations:** ^1^ College of Ethnic Medicine, Yunnan University of Chinese Medicine, Kunming, Yunnan, China; ^2^ College of Chinese Medicine, Yunnan University of Chinese Medicine, Kunming, Yunnan, China; ^3^ College of Basic Medical Sciences, Yunnan University of Chinese Medicine, Kunming, Yunnan, China

**Keywords:** DaiTongXiao, gout nephropathy, traditional Chinese medicine, TLR4/MyD88/NF-κB pathway, mechanism research

## Abstract

**Introduction:** Gouty nephropathy (GN) arises from factors like excessive purine intake, metabolic disorders or abnormal synthesis, and uric acid hypersaturation in the blood, leading to urate crystal deposition in kidney tissue. DaiTongXiao (DTX) is a remedy used by the Dai people of China. It shows efficacy in lowering uric acid levels and exhibits anti-inflammatory and kidney-protective properties.

**Methods:** A GN rat model was induced using adenine and potassium oxonate. Following DTX administration, various parameters were assessed in urine, serum, and kidney tissue. Western blot analysis evaluated TLR4/MyD88/NF-κB signaling proteins, while immunofluorescence examined NF-κB nuclear expression.

**Results:** DTX treatment improved kidney morphology, increased body weight, and kidney index and enhanced urinary levels of blood urea nitrogen (Bun), 24-h urinary protein, uric acid (UA), and allantoin in GN rats, reducing UA, Bun, creatinine (Cre), cystatin C (CysC), serum amyloid A (SAA), α1-microglobulin (MG), and β2-MG in serum analysis. Renal tissue assessments showed decreased xanthine oxidase (XOD), hydroxyproline (Hyp), α-smooth muscle actin (α-SMA), and collage type Ⅳ (COL-Ⅳ). Kidney damage severity was notably reduced. DTX lowered serum inflammatory factors like interleukin (IL) −18, tumor necrosis factor-α (TNF-α), C-reactive protein (CRP), transforming growth factor-β1 (TGF-β1), and IL-1β in the rat serum, reducing chemokine monocyte chemoattractant protein-1 (MCP-1) and adhesion factor vascular cell adhesion molecule-1(VCAM-1). Western blotting demonstrated the downregulation of TLR4/MyD88/NF-κB pathway proteins, and immunofluorescence revealed reduced NF-κB expression in renal tissue.

**Discussion:** DTX exhibits significant anti-GN effects by modulating TLR4/MyD88/ NF-κB pathway protein expression, reducing inflammatory factor release, and inhibiting GN progression.

## Graphical Abstract

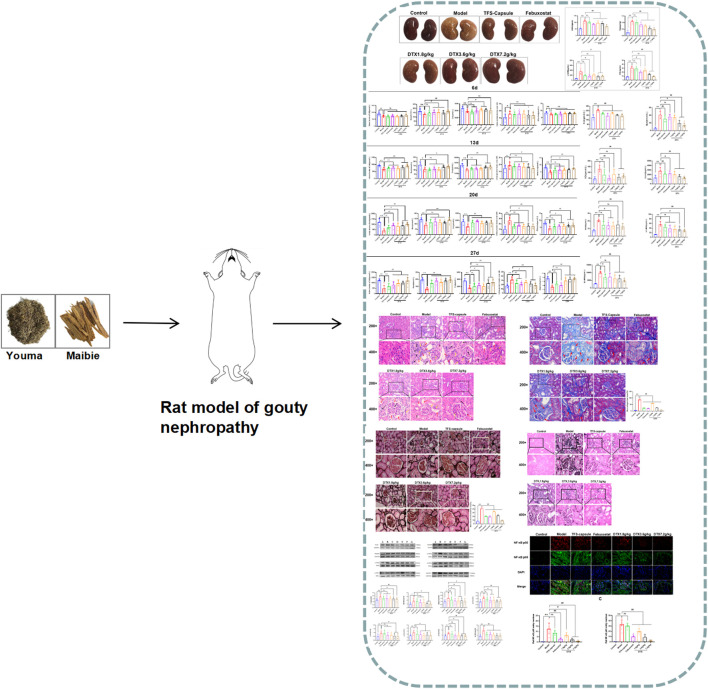

## Highlights

- Revealing the effects of DTX on rats with gout nephropathy.
- Revealing that DTX has significant ameliorative effects on gout nephropathy.
- Revealing that DTX can improve gout nephropathy by inhibiting inflammatory response through the TLR4/MyD88/NF-κB pathway.

## 1 Introduction

Currently, gouty nephropathy (GN) prevalence has been rising annually. Findings show that clinically, around 20% of gout patients have chronic progressive renal lesions, while autopsy confirms renal lesions in up to 100% of gout cases. After 5–10 years, the condition worsens, glomerular function is impaired, and renal insufficiency occurs, leading to GN, attributable to shifts in life quality and dietary habits spurred by socioeconomic advancements (Mei et al., 2022). GN is a condition characterized by aberrant blood uric acid (UA) production or urate excretion formed in the kidney medulla, causing local inflammation and interstitial fibrosis in the kidney, also known as hyperuricemia nephropathy (Pan et al., 2021). GN is a chronic renal lesion caused by abnormal purine metabolism in the body, which leads to long-term supersaturation of blood uric acid and the deposition of excessive urate crystals in the kidneys. The pathological mechanism of the occurrence of GN may be related to the occurrence of inflammatory reactions, endothelial dysfunction, fibrosis, proliferation of vascular smooth muscle cells, activation of the renin–angiotensin system, and glomerular arteriosclerosis, among other processes. Clinical symptoms are often manifested as increased nocturia due to decreased urinary concentrating function, and manifestations of renal insufficiency, such as hypertension, edema, and anemia, appear in the late stage due to decreased glomerular filtration function. Currently, specific therapeutic agents for GN are lacking, and symptomatic treatment remains the primary approach in clinical practice. The commonly used drugs include allopurinol, febuxostat, and colchicine, all aimed at inhibiting the synthesis of uric acid (2012). However, all the aforementioned drugs have significant adverse effects; for instance, long-term application of allopurinol can cause severe liver damage (Grace and Luke, 2022), febuxostat may trigger generalized rash (H-Y et al., 2015), and colchicine is linked to cardiomyopathy and cytopenia upon prolonged application (Prasanna et al., 2020). Existing treatments have major limitations in addressing both the occurrence and development of GN. Exploring traditional prescriptions for medicines with potent efficacy and fewer adverse reactions holds profound significance. Ethnomedicines, predominantly composed of natural remedies, have demonstrated distinct advantages in combating GN.

DaiTongXiao (DTX) is a traditional remedy widely employed by the Dai community in China for treating gout, boasting a history spanning over 2,500 years (ZHENG et al., 2022). In contemporary practice, DTX is utilized to alleviate conditions stemming from elevated uric acid levels, such as gout, as detailed in the 21st-century Dai Medical Clinical Science undergraduate textbook (Wu et al., 2022; ZHENG et al., 2022). Clinical investigations involving 29 gout patients treated with DTX revealed significant improvement in 25 cases, with 4 cases showing partial recovery, yielding an overall 86.2% efficacy rate (Feng, 2015). Comprising You Ma and Mai Bie in a 3:1 ratio, the DTX formulation includes whole grass of *Elsholtzia rugulosa* Hemsl. (You Ma) in the Lamiaceae family, supported by modern research, with main components including triterpenoids, flavonoids, sterols, and their glycosides. These constituents endow it with antipyretic and detoxifying properties as well as antioxidant and uric acid-lowering effects (Zhou and Chen, 2021). Mai Bie, derived from pine (*Pinus tabuliformis* Carrière.) nodules and branch nodes, predominantly contains flavonoids, volatile oils, etc. (Zhang, 2009; Zhang et al., 2010). Renowned for its anti-inflammatory and antioxidant attributes, Mai Bie offers therapeutic potential (Shi et al., 2014). It has been shown that α-pinene and β-pinene, the active components in volatile oils, can significantly reduce the levels of inflammatory factors such as IL-6, IL-1β, and TNF-α, thus exerting anti-inflammatory effects (Jena et al., 2022). In addition, the flavonoid component of apigenin can play a role in protecting the kidneys by lowering uric acid levels and regulating the uric acid transporter group (Liao et al., 2016).

Prior research has demonstrated that DTX markedly diminishes serum blood urea nitrogen (Bun), creatinine (Cre), and uric acid (UA) in a mouse model of yeast-induced hyperuricemia, thus exerting a protective influence on renal function. Furthermore, in the monosodium urate (MSU)-induced gout rat model, DTX effectively suppresses pro-inflammatory cytokines, including tumor necrosis factor-α (TNF-α), interleukin (IL)-1β, and IL-6 secretion, mitigating inflammatory damage and exhibiting anti-inflammatory properties [9]. Further study found that through MSU injection to replicate the gout rat model, DTX substantially attenuates NLRP3 protein expression and ameliorates gout symptoms (Feifan et al., 2023). Recent research indicates that GN pathogenesis may be governed by the TLR4/MyD88/NF-κB pathway. TLR4 is situated on the cell membrane as an initiating factor of the TLR4/MyD88/NF-κB pathway. Upon recognition of MSU, pivotal adapter protein MyD88 triggers NF-κB and NLRP3 inflammasome activation. Subsequently, numerous inflammatory mediators implicated in the inflammatory immune response, such as TNF-α and IL-1β/-6, undergo further activation and regulation, instigating an inflammatory cascade amplification effect. This process culminates in GN progression. Thus, targeting the TLR4/MyD88/NF-κB pathway may represent a promising therapeutic approach for GN (Hu et al., 2020; Hosoyamada, 2021).

In this investigation, the combination of adenine and potassium oxonate was used to induce the rat GN model. The study aimed to investigate whether the mechanism underlying DTX’s anti-GN effects involves TLR4/MyD88/NF-κB pathway modulation, thereby diminishing inflammatory factor release and ultimately enhancing kidney protection.

## 2 Materials

### 2.1 Drugs

DTX, formulated with a 3:1 ratio of You Ma and Mai Bie, includes herbs sourced from the Yunnan Medicinal Materials Market in China and authenticated as genuine by Professor Feng Deqiang from the Yunnan University of Traditional Chinese Medicine (Table 1). Active ingredient content adhered to standards outlined in Chinese Pharmacopoeia and local regulations. Previous investigations utilized ultraviolet spectrophotometry and gas chromatography to analyze active constituents present in DTX.

**TABLE 1:** Drug composition of DTX.

Local name	Botanical name	Species name	Authoritative name
You Ma	*Elsholtzia rugulosa* Hemsl	Lamiaceae	-
Mai Bie	*Pinus Tabuliformis* Carrière	Pinaceae	Pini lignum nodi

We precisely weighed 2 g of DTX extract and subjected it to reflux extraction with 50% ethanol for 2 h in a 50 mL volume. After cooling, we utilized 50% ethanol to compensate for weight loss and filtration and obtained filtrate as a test solution. The absorbance value was measured at 510 nm, and the total flavonoid content of the DTX extract was subsequently calculated. The findings revealed a strong linear relationship between total flavonoid concentrations in DTX and corresponding absorbance within 2.4–6.4 μg/mL (R^2^ = 0.9993). The average recovery rate was 95.7% (RSD = 1.95%), and the total flavonoid content in DTX was determined to be 677.3 μg/mL.

**FIGURE 1:**
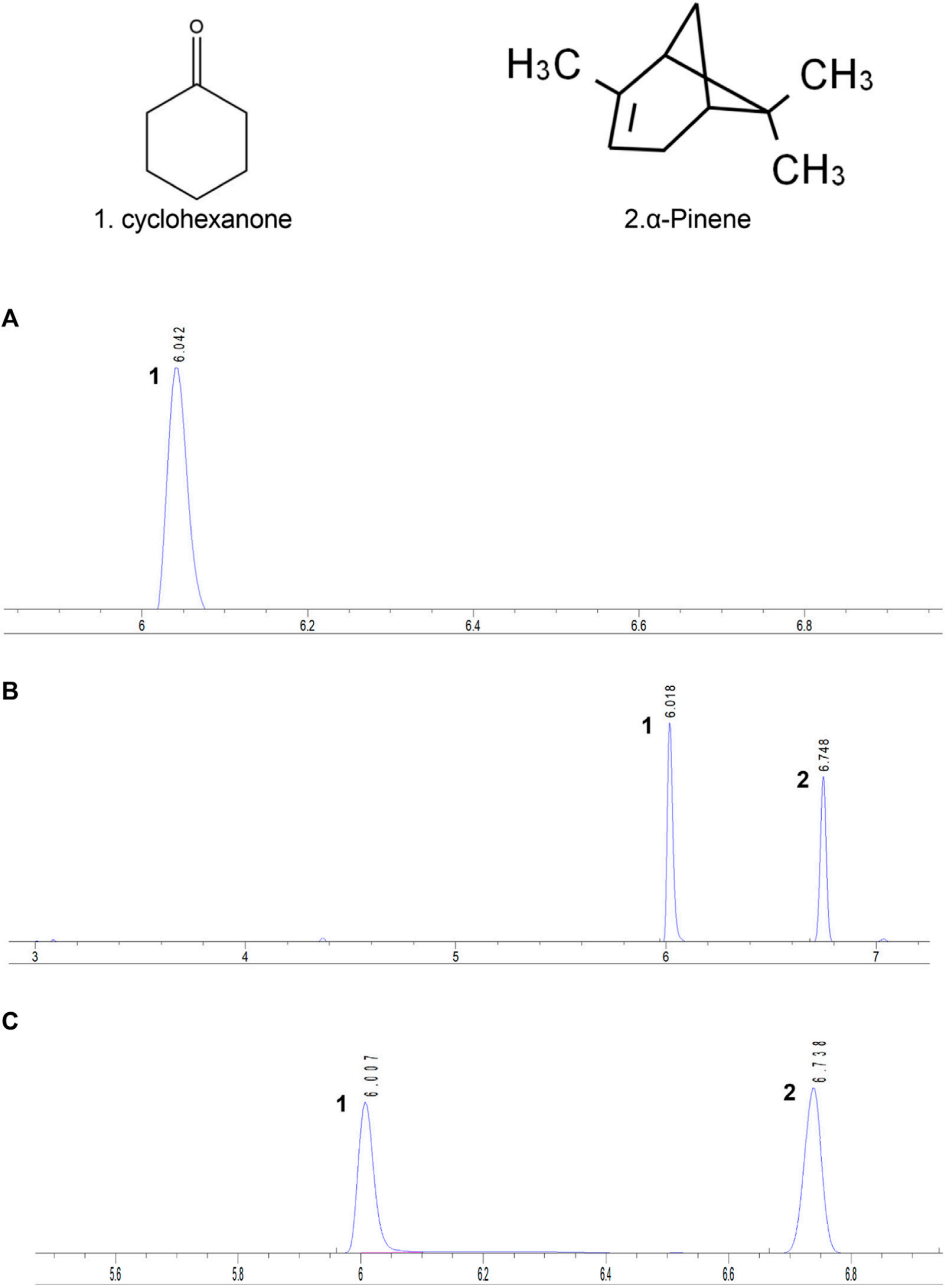
Each sample’s gas chromatogram. **(A)** Control, **(B)** standard, and **(C)** testing solutions. 1. Cyclohexanone. 2. α-Pinene (Liu et al., 2024).

Furthermore, the DTX compound encompasses various volatile oil components, among which α-pinene stands out for its analgesic and anti-inflammatory capabilities, rendering it an effective ingredient for gouty arthritis treatment. Approximately 30 g of the DTX extract was accurately weighed, and DTX volatile oil of 0.1 mL was extracted according to the determination method outlined in the Appendix of the 2010 edition of the People’s Republic of China Pharmacopoeia (method A). The extracted volatile oil was then quantitatively transferred into a 5-mL vial using n-hexane and diluted to the appropriate scale. Subsequently, the cyclohexanone internal standard solution (100 μL) (prepared by diluting 2 mL of cyclohexanone with n-hexane to an appropriate scale) was accurately added, followed by thorough shaking. The results demonstrated a robust linear relationship between the α-pinene injection amount within 0.08–0.4 mg (R^2^ = 0.9994). The average recovery rate was determined to be 101.11% (RSD = 4.26%). The α-pinene content in DTX was quantified at 665.7 mg/mL (Figure 1) (Liu et al., 2024).

The TFS capsule, purchased from Shaanxi Panlong Pharmaceutical Group Co., Ltd (Batch No. 20220503), is renowned for its efficacy in rectifying purine metabolism disorders. It aids in restoring hepatic and renal uric acid metabolism, thereby reducing renal function impairment (Sun et al., 2023). Febuxostat was taken as a xanthine oxidase suppressor, purchased from Jiangsu Wanbang Biochemical Pharmaceutical Group Limited Liability Company (Batch No. 62212754). It impeded uric acid synthesis, thus effectively lowering serum uric acid levels while simultaneously exerting anti-inflammatory effects to reduce renal injury (Kraev et al., 2023). Both TFS capsule and febuxostat were employed as positive control drugs in this study.

To investigate DTX’s impact on the kidneys of rats with GN, we induced a GN rat model using the combination of the uricogenic agent adenine and the uricase inhibitor potassium oxonate. The adenine-induced GN model primarily elevates adenine intake, resulting in increased uric acid levels and uric acid crystal formation in the kidneys, thus precipitating GN. This model exhibits a certain degree of self-healing within a short period because rats possess uricase that further metabolizes uric acid into allantoin, which is then excreted from the body. To mimic the internal biological environment in humans more accurately, this experiment introduced potassium oxonate, an inhibitor of urate oxidase, alongside adenine modeling. This addition inhibits the activity of urate oxidase, preventing the oxidative breakdown of uric acid and resulting in the accumulation of uric acid in the body, ultimately inducing GN (Zhang et al., 2023a).

### 2.2 Reagents

UA, Cre, Bun, urinary protein, xanthine oxidase (XOD), and hydroxyproline (Hyp) kits (Lot Nos C012-2-1, C011-2-1, C035-2-1, C013-2-1, A002-1-1, and A030-2-1, respectively) were procured from Nanjing Jiancheng Bioengineering Institute. Allantoin (Lot No. YJ059176) was sourced from Shanghai Yase Biomedical Technology Co., Ltd. Cystatin C (CysC), α1-microglobulin (MG), β2-MG, IL-1β, IL-18, TNF-α, TGF-β1, and MCP-1 kits (Lot Nos JYM0259Ra, JYM0125Ra, JYM0066Ra, JYM0419Ra, JYM0650Ra, JYM0635Ra, JYM0527Ra, and JYM0495Ra, respectively) were sourced from Wuhan Genome Biotechnology Co., Ltd. Serum amyloid A (SAA), α-smooth muscle actin (α-SMA), collage type Ⅳ (COL-Ⅳ), vascular cell adhesion molecule-1 (VCAM-1), and CRP (Lot Nos A-R00411A, A-R01271B, A-R01941A, A-R02062A, and A-R00073A, respectively) were purchased from Aidea Technology (Beijing) Co., Ltd. TLR4, MyD88, HMGB1, p-IKKα, IKKα, p-IKKβ, IKKβ, p-IKKγ, IKKγ, NLRP3, GAPDH, β-actin antibody (Lot Nos AF7017, AF5195, AF7020, AF3012, AF6014, AF3010, AF6009, AF3496, AF6495, AF4620, AF7021, and AF7018, respectively) were purchased from Affinifity Biosciences Co., Ltd. p-IκBα, IκBα antibody (Lot Nos CST2859, CST9242, respectively) were purchased from Cell Signaling Technology Co., Ltd.

### 2.3 Experimental animals

Seventy SPF-grade male SD rats, aged 8 weeks and with a body mass of 200 ± 20 g, were purchased from Sibeifu (Beijing) Biotechnology Co., Ltd. (License No: SCXK (Beijing) 2019–0010). They were housed at a feeding temperature of 23 ± 2°C and humidity of 50 ± 5 %, with free access to food and water. Animal management and corresponding experiments were carried out following the Animal Welfare and Management Act and Guidelines for the Care and Use of Laboratory Animals of the National Research Council of the United States and checked and approved by the Ethics Committee of the Yunnan University of Chinese Medicine (Approval number: R-062023094). Protocols for assays comply with the Declaration of Helsinki and ethical standard guidelines.

## 3 Methods

### 3.1 DTX preparation

Preparation of DTX: 12 times the volume of water was added to the prescribed botanical medicine, soaked for 0.5 h, and then transferred to a flask. A condensing tube was attached, and extraction was performed three times. Each extraction used 12 times the amount of pure water, each for 1 h (100°C). The DTX water extracts from the three extractions were combined. The solution was filtered through gauze and freeze-dried into an extract. The DTX extract was diluted using distilled water to concentrations of 0.72, 0.36, and 0.18 g raw drug/mL as DTX high/medium/low-dose solutions, respectively.

### 3.2 Identification of the composition of DTX

#### 3.2.1 UHPLC-OE-MS analysis

##### 3.2.1.1 Sample handling

A measure of 10 mL of DTX was centrifuged at 4°C at 12,000 rpm for 15 min. Using a pipette, 300 μL of the supernatant was accurately absorbed, and 1,000 μL of the extraction solution [methanol: water (4:1, v/v)] containing the internal standard of the isotope label was added. The supernatant was vortexed for 30 s, subjected to ice water bath ultrasound for 5 min, stored in the refrigerator at −40°C for 1 h, and then centrifuged at 4°C at 12,000 rpm for 15 min. Then, the supernatant was filtered through a 0.22-μm microporous filter membrane into a sample vial and was tested on the machine.

##### 3.2.1.2 Analyzing conditions

Chromatographic conditions: A Phenomenex Kinetex C18 Column (2.1 mm × 100 mm, 2.6 μm) was used. The mobile phase consisted of two components: A, the aqueous phase containing 0.01% acetic acid and B, isopropanol: acetonitrile (1:1, v/v).

Mass spectrometry conditions: Sheath gas flow rate, 50 Arb; auxiliary gas flow rate, 15 Arb; capillary temperature, 320°C; full ms resolution. 60,000; MS/MS resolution, 15,000; collision energy, SNCE 20/30/40; and spray voltage, 3.8 kV (positive) or −3.4 kV (negative).

#### 3.2.2 GC-MS analysis

##### 3.2.2.1 Sample handling

DTX samples were precisely absorbed from 100-μL to 10-mL volumetric bottles, passed through a 0.22-μm microporous filter membrane, placed in the container, sealed well, and adsorbed for 50 min in a solid-phase microextraction column at a constant temperature of 60°C before directly being injected for analysis.

##### 3.2.2.2 Analyzing conditions

Gas phase condition: Agilent HP-5MS Quartz Capillary Column (30 m × 0.25 mm × 0.25 μm) was used. Column temperature: starting temperature, 40°C; program temperature, 3°C/min to 80°C, 5°C/min to 280°C, and 10 min; inlet temperature, 250°C; and column front pressure, 100 kPa. The carrier gas was high-purity helium, the sample size was 1 μL, the shunt ratio was 2:1, and the flow rate was 1.0 mL/min.

Mass spectrum conditions: ionization mode, EI; electron energy, 70°eV; transmission line temperature, 250°C; ion source temperature, 230°C; quadrupole temperature, 150°C; scan quality range, 35–500. Wiley7n.l and NIST98.L standard spectrum libraries were used for qualitative search.

### 3.3 Drug dosage

To establish rat dosages equivalent to clinical DTX human dosages, a conversion based on the surface area was conducted. The clinical human dosage of DTX was a raw drug of 20 g per day. In animal experiments, doses equivalent to 1, 2, and 4 times the clinically relevant human dosage of DTX were chosen. These corresponded to small-dose (raw drug 1.8 g/kg), medium-dose (3.6 g/kg), and large-dose groups (7.2 g/kg). Doses were decided by surface area conversion between rats and humans (Formula 1). Furthermore, based on literature references, the TFS capsule was administered at 600 mg/kg (Li et al., 2022), while febuxostat was administered at 3.6 mg/kg (Shen et al., 2020).

Dosage for rats=X mg/kg×70 kg×0.018/200 g. (1)

Formula 1 represents human and rat body surface area conversion formula. X is the clinical dose for humans, 70 kg is the weight of humans, 0.018 is the conversion factor, and 200 g is the weight of rats.

### 3.4 Drug preparation

Preparation of adenine + potassium oxazinate solution: Adenine (100 mg) and potassium oxazinate (200 mg) powder were taken, dissolved in the 0.5% CMC-Na solution (1000 rpm/min, 20 min), and then adenine + potassium oxazinate suspension solution was obtained.

Preparation of the TFS capsule solution: Goufengshu powder of 600 mg was precisely weighed using the precision electronic balance. The powder was dissolved in 10 mL of double-steamed water (at 1000 rpm/min for 10 min) to prepare a TFS capsule solution of 60 mg/mL.

Preparation of the febuxostat solution: Using a precision electronic balance, febuxostat powder of 3.6 mg was weighed. The powder was dissolved in 10 mL of distilled water (at 1000 rpm/min for 15 min) to obtain a febuxostat solution at 0.36 mg/mL.

### 3.5 Modeling, grouping, and administration

**FIGURE 2:**
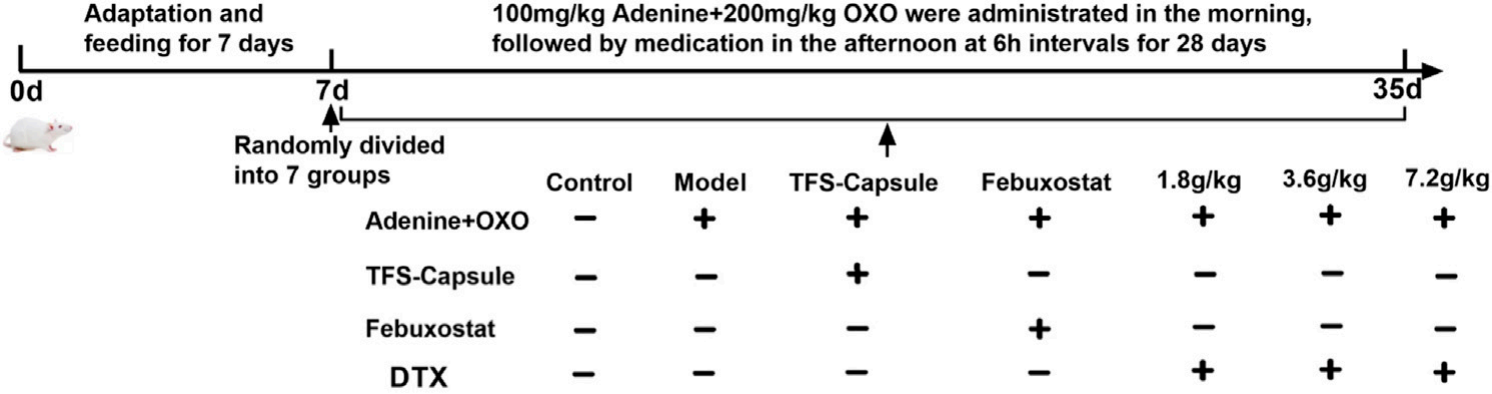
Replicating the time axis of the GN rat model.

A total of 70 healthy male SD rats weighing 180–220 g underwent random assignment into 8 cohorts following 1 w of acclimatization: control, model, FSS capsule, febuxostat, DTX, 1.8 g raw drug/kg, 3.6 g raw drug kg, and 7.2 g raw drug/kg groups. Except for the control group, the model was induced by intragastric administration of 100 mg/kg adenine + 200 mg/kg potassium oxazinate (solvent 0.5% CMC-Na) with intragastric 10 mL/kg across all other cohorts. After a 6 h interval, each group received corresponding intragastric drug administration according to Table 2. Rats in control and model cohorts received the administration of distilled water (10 mL/kg) once daily for 28 days. See Figure 2.

**TABLE 2:** Dosage of animals in each group.

Group	Dosage of administration
Control	Equivalent distilled water
Model	Equivalent distilled water
TFS capsule	600 mg/kg
Febuxostat	3.6 mg/kg
DTX small dose	1.8 g raw drug/kg
DTX medium dose	3.6 g raw drug/kg
DTX high dose	7.2 g raw drug/kg

### 3.6 Routine state observation of rats

The body weights of the rats were measured and recorded weekly throughout the experiment. After the final induction, each group of rats was subjected to a comprehensive assessment, including the observation and recording of hair quality using a gross morphology detection scale and the observation and recording of the rat’s mental state, morphology, and activity level using a behavioral rating scale.

### 3.7 DTX impact on UA, Bun, 24 h urinary protein, and allantoin in the urine of GN rats

On the 6th, 13th, 20th, and 27th days, rats from each group were placed individually in a metabolic cage for 24 h. After a 30-min settling period, urine samples were centrifuged (at 4°C and 4000 rpm for 10 min). After obtaining the supernatant, it was stored at −80°C for 24 h, and UA, Bun, 24-h urinary protein, and allantoin were assessed and quantified following the instructions provided with the respective kits.

### 3.8 DTX effects on serum UA, Cre, Bun, CysC, SAA, α1-MG, and β2-MG levels in GN rats

Following final administration, rats were deprived of water for 12 h and then weighed. For anesthesia, each rat group received an intraperitoneal injection (2 mL/kg) of 2% pentobarbital sodium. Once anesthetized, rats were secured onto the rat plate, with blood samples collected from the abdominal aorta. The blood samples were allowed to stand at room temperature for 1 h before centrifugation to separate the serum (at 4°C and 4000 rpm for 10 min). The separated serum samples were stored at −80°C. UA, Cre, Bun, CysC, SAA, α1-MG, and β2-MG levels in the serum were determined following the instructions provided with the respective kits.

### 3.9 Effects of DTX on the renal index in GN rats

Rat’s kidney was carefully extracted, rinsed, and filter paper-dried. Its weight was recorded, and subsequently, the kidney index was calculated utilizing the formula given below. Left kidney tissues were fixed with 50 mL of 4% neutral formaldehyde at room temperature for 24 h for subsequent pathology. Meanwhile, right kidney tissues were preserved for afterward use (−80°C).

$$\text{Renal index}=\frac{\text{Bilateral}\;\text{renal}\;\text{mass}}{\text{Body}\;\text{mass}\;\text{of}\;\text{rats}}\times 100\%.$$

### 3.10 Effects of DTX on renal XOD, Hyp, α-SAM, and COL-Ⅳ levels in GN rats

Rat kidney tissue weighing 100 mg was taken and homogenized with normal saline (900 μL) using a high-speed freezing grinder for high-frequency homogenization. Following homogenization, the mixture was centrifuged at 4°C and 5,000 rpm for 15 min, yielding the supernatant. XOD, Hyp, α-SAM, and COL-Ⅳ in renal tissue homogenates were then measured.

### 3.11 Renal histopathological examination

The left kidney tissue was placed in 50 mL of 4% paraformaldehyde solution and fixed at room temperature for 24 h. Subsequently, paraffin sections were prepared, followed by dewaxing and dehydration processes. Staining procedures, including H&E, Masson’s trichrome, periodic acid–Schiff–methenamine (PASM), and Gomori staining, were sequentially performed. Renal histopathology was observed using the automatic digital biopsy scanner.

In each group, evaluations were made regarding inflammatory infiltration, collagen fiber density, basement membrane thickening, and urate deposition.

For H&E staining, the degree of inflammatory infiltration was assessed in this study using a scoring system, with no lesions scored as 0, mild or very little scored as 1, mild or little scored as 2, moderate scored as 3, severe scored as 4, and very severe scored as 5.

For Masson’s staining, the situation of collagen fiber density was analyzed in this study using Image-Pro Plus 6.0 software, and the same blue color was selected as a uniform criterion for determining the positivity of all photographs, and each photograph was analyzed to derive the ratio of positive staining to the entire area of the tissue in each photograph, i.e., the percentage of the area that was positive (%).

For PASM staining, the basement membrane thickening was analyzed in this study using Image-Pro Plus 6.0 software, and the same black color was selected as a uniform criterion for determining the positivity of all photographs, and each photograph was analyzed to derive the ratio of positive staining to the entire area of the tissue in each photograph, i.e., the percentage of the area that was positive (%).

For Gomori staining, urate deposition was analyzed in this study using Image-Pro Plus 6.0 software, and the same black color was selected as a uniform criterion for determining the positivity of all the photographs, and each photograph was analyzed to derive the ratio of positive staining to the entire area of the tissue in each photograph, i.e., the percentage of the area that was positive (%).

### 3.12 Effects of DTX on inflammatory cytokines, chemokines, and adhesion factors

Rat serum samples were collected, and serum MCP-1, IL-18, VCAM-1, CRP, TNF-α, IL-1β, and TGF-β1 were determined via Elisa assays.

### 3.13 DTX regulatory effects on renal tissue proteins were quantified by Western blotting

HMGB1, p-IKKγ/IKKγ, MyD88, NLRP3, TLR4, p-IκBα/IκBα, p-IKKα/IKKα, and p-IKKβ/IKKβ proteins in the TLR4/MyD88/NF-κB pathway were detected by Western blotting. Rat kidney tissue of uniform size was clipped on ice, and an appropriate amount of lysate was added according to the weight of the kidney: lysate (prepared by RIPA: PMSF = 100:1) = 1:10. Rat kidney tissue was homogenized using a high-speed freezing grinder at high frequency, followed by centrifugation (at 12,000 rpm for 10 min) to obtain protein supernatant. BCA was utilized to measure the total protein concentration in each sample. Subsequently, each sample was adjusted to 2 μg/μL using cracking solution, 5X loading buffer, and sample leveling. The samples were then boiled at 95°C in a metal thermostat for 5 min and rapidly cooled on ice. After subpackaging, they were stored at −80°C. Constant pressure electrophoresis (at 180 V for 40 min) was performed. Constant pressure transfer was done (at 110 V for 70 min). Milk was enclosed (at room temperature for 2 h). TLR4 (1:2000), MyD88 (1:1000), HMGB1 (1:1000), p-IKKα (1:500), IKKα (1:500), p-IKKβ (1:500), IKKβ (1:500), p-IKKγ (1:500), IKKγ (1: 500), p-IκBα (1:500), IκBα (1:500), NLRP3 (1:2000), and GAPDH (1:500) were enclosed in a refrigerator at 4°C overnight, and the goat anti-rabbit secondary anti-antibody (1:500) was added and enclosed (at room temperature for 1 h). Targeted protein/internal reference protein bands’ OD value represents target protein expression.

### 3.14 NF-κB protein nucleation was observed using fluorescence immunoassay

Dehydrated rat kidney tissue was incubated with NF-κB p50 (1:1000) and NF-κB p65 (1:1000). NF-κB p50 and NF-κB p65 protein nucleation were observed using a fluorescence microscope after restaining the cell nucleus with DAPI and FITC (1:400) for visualization with green light.

### 3.15 Molecular docking

The structure of chemical compounds (*PDB/mol2 format) was downloaded from the TCMSP and PubChem databases. From the PDB database, the 3D structure of the target protein (PDB format) was downloaded for future molecular docking. PyMOL software was used to dehydrate and remove the ligand of the active center. The target protein was hydrogenated, and small drug molecule rotation bonds were established using AutoDock software and then collected in the PDBQT format. AutoDock was used to find optimal conformation. Finally, the detailed docking information was analyzed and visualized using PyMOL software.

### 3.16 Statistical analysis

Data analysis was performed using SPSS software (Ver. 26.0). One-way ANOVA was employed for multiple group comparisons when data followed a normal distribution. *Post hoc* analysis was performed using the LSD method for the homogeneity of variances, while Dunnett’s T3 method was applied for multiple comparisons. Non-parametric tests were employed for datasets without demonstrating normal distribution. *p* < 0.05 was considered statistically significant.

## 4 Results

### 4.1 Results of UHPLC-OE-MS

**FIGURE 3:**
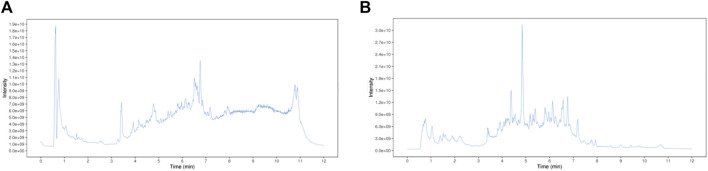
DTX chemical composition total ionogram. **(A)** Positive ion mode and **(B)** negative mon mode.

In this study, the chemical composition of DTX in positive- and negative-ion modes was analyzed via UHPLC-Q-TOF MS. The mass spectrometry data were processed and analyzed using MS-DIAL software to automatically match compound fragment information and identify known components to a compound database. As a result, a total of 189 chemical components were initially identified by comparing retention times and fragmentation patterns with references, and the results are shown in Supplementary Table S1; Figure 3.

### 4.2 Results of GC-MS

**FIGURE 4:**
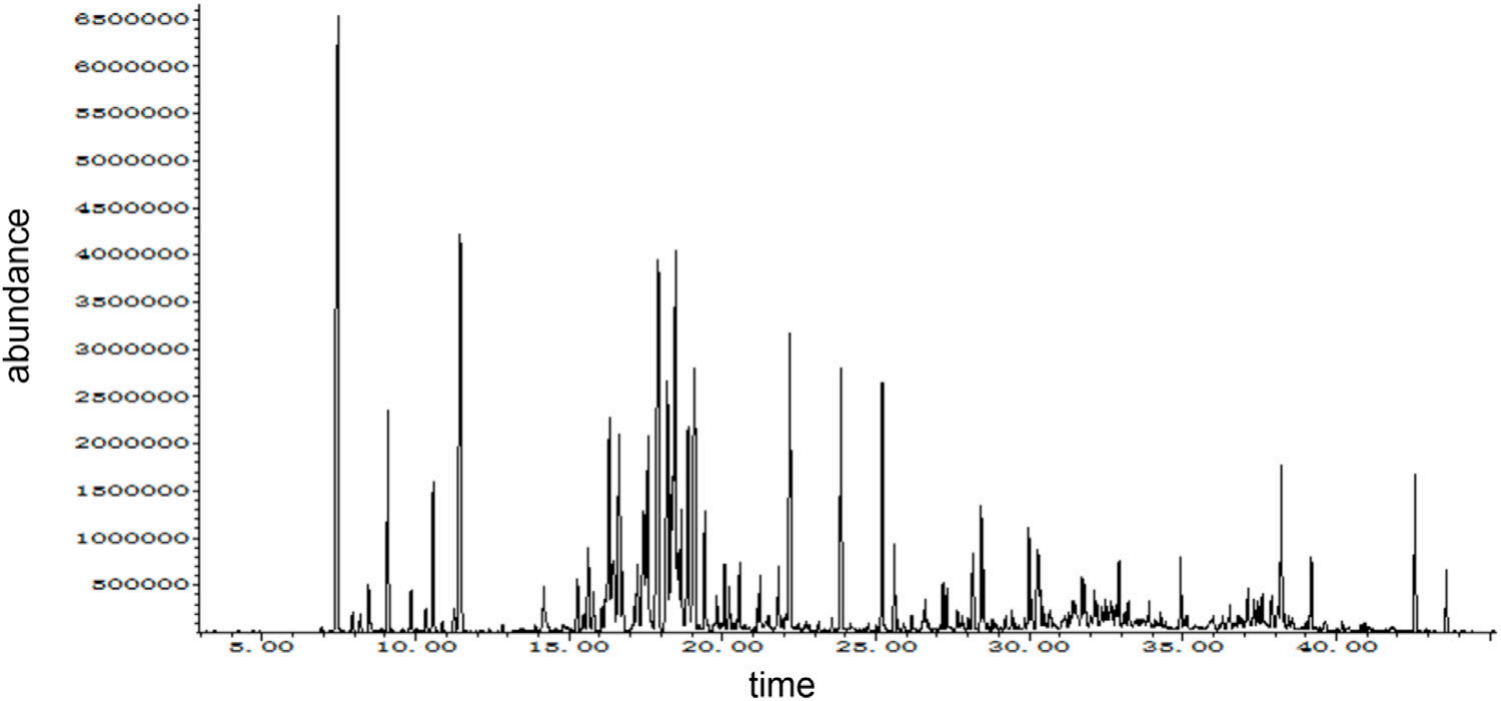
GC-MS ion map.

The composition of DTX analyzed in this study was performed using GC-MS. The mass spectrometry data were processed and analyzed using Wiley7n.l and NIST98.L in order to automatically match compound fragment information and identify known constituents with the compound database. As a result, a total of 24 chemical components were initially identified by comparing the retention times with the references, and the results are shown in Table 3; Figure 4.

**TABLE 3:** Results of GC-MS determination.

No.	t_R_/s	Ingredient	Formula
1	7.47	α-Pinene	C_10_H_16_
2	8.47	Benzaldehyde	C_7_H_6_O
3	9.1	β-Pinene	C_10_H_1__6_
4	9.85	β-Myrcene	C_10_H_1__6_
5	10.57	3-Carene	C_10_H_1__6_
6	11.26	1-methoxy 4-methyl 2, 1-methylethyl benzene	C_11_H_16_O
7	11.46	Sabinene	C_10_H_16_
8	14.18	α-Terpinene	C_10_H_16_
9	15.27	(+)-Fenchyl alcohol	C_10_H_18_O
10	15.63	2-Cyclohexen-1-ol, 1-methyl-4-(1-methylethyl)-, trans-	C_10_H_18_O
11	15.79	3-Cyclopentene-1-acetaldehyde, 2,2,3-trimethyl-, (R)-	C_10_H_16_O
12	16.19	Bicyclo[3.1.1]heptan-3-one, 2,6,6-trimethyl-	C_10_H_16_O
13	17.44	Borneol	C_10_H_18_O
14	17.91	3-Cyclohexen-1-ol, 4-methyl-1-(1-methylethyl)-, acetate	C_12_H_20_O_2_
15	18.35	Benzenemethanol, α,α,4-trimethyl-	C_10_H_14_O
16	18.48	3-Cyclohexene-1-methanol, alpha, alpha, 4-trimethyl-, 1-acetate, (1R)-	C_12_H_20_O_2_
17	19.43	2-Cyclohexen-1-ol, 2-methyl-5-(1-methylethenyl)-, acetate, cis-	C_12_H_18_O_2_
18	19.82	cis-Carveol	C_10_H_16_O
19	20.07	Benzaldehyde, 4-(1-methylethyl)-	C_10_H_12_O
20	20.22	2-Cyclohexen-1-one, 2-methyl-5-(1-methylethenyl)-, O-methyloxime, (+)-	C_11_H_17_NO
21	20.56	2-Cyclohexen-1-one, 3-methyl-6-(1-methylethyl)-	C_10_H_16_O
22	21.82	Benzenemethanol, 4-(1-methylethyl)-, acetate	C_12_H_16_O_2_
23	23.87	Phenol, 2-methoxy-3-(2-propenyl)-	C_10_H_12_O_2_
24	25.21	(+)-Longifolene	C_15_H_24_
25	25.60	β-Caryophyllene	C_15_H_24_
26	27.18	+α-Amorphene	C_15_H_24_
27	27.31	Germacrene D	C_15_H_24_
28	28.15	Phenol, 2,4-bis-(1,1-dimethylethyl), TMS	C_17_H_30_OSi
29	28.43	(+)-delta-Cadinene	C_15_H_24_
30	28.52	Phenol, 2-methoxy-4-(2-propenyl)-, acetate	C_12_H_14_O_3_
31	29.98	β-Caryophyllene oxide	C_15_H_24_O
32	30.28	Hexadecane	C_16_H_34_
33	31.72	α-Cadinol	C_15_H_26_O
34	32.12	Caryophyllenol-II	C_15_H_24_O
35	32.66	Heptadecane	C_17_H_36_
36	32.91	Pentadecane	C_15_H_32_
37	34.93	Octadecane	C_18_H_38_
38	35.99	Heneicosane	C_21_H_44_
39	39.19	Isopimaradiene	C_20_H_32_

### 4.3 DTX improved general conditions of GN rats

Throughout the experiment, control rats exhibited robust activity, maintained normal hair color, consumed their diets as usual, and showed no adverse signs. Conversely, rats in the model group displayed reduced activity, lackluster hair color, and lethargy. In comparison, rats administered DTX at 1.8, 3.6, and 7.2 g/kg exhibited improved mental states, restored hair luster, normal dietary patterns, and regular stool consistency when compared to the model group.

### 4.4 DTX improved kidney morphology in GN rats

**FIGURE 5:**
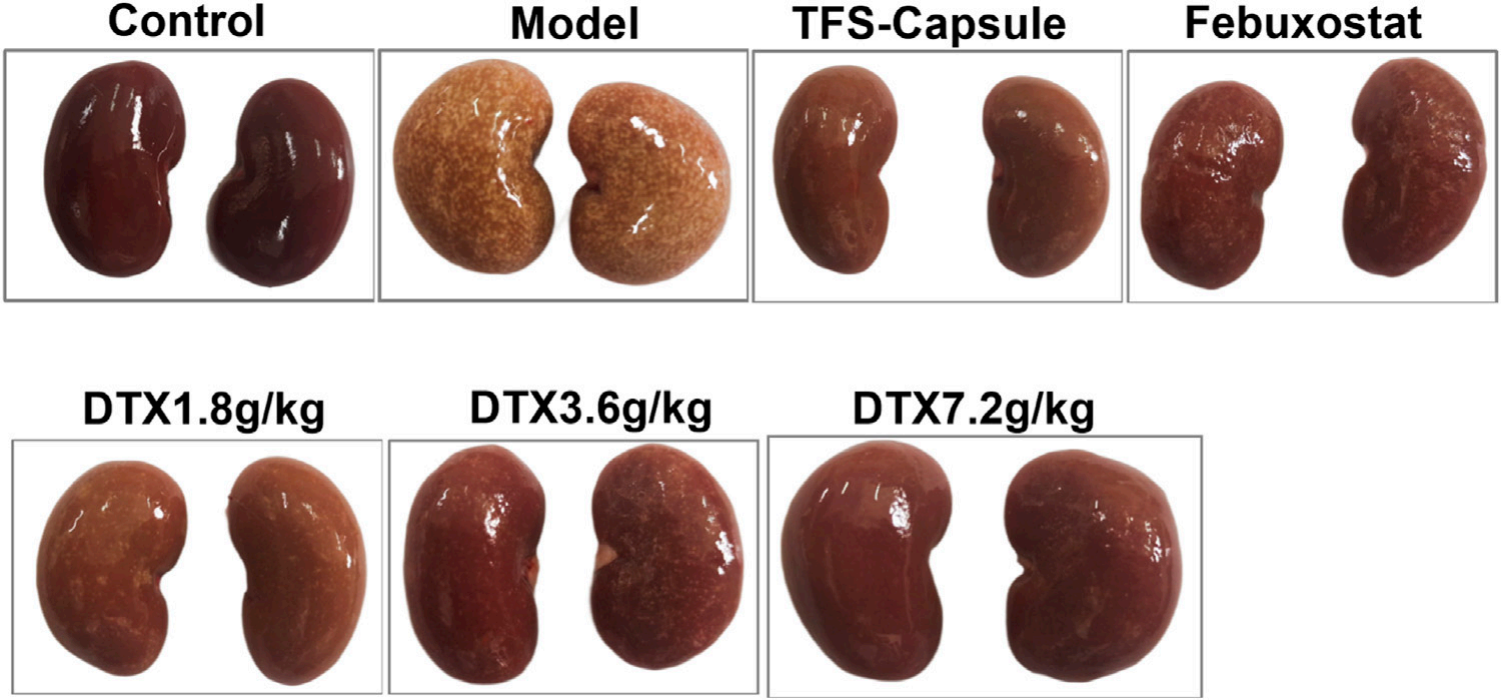
DTX improved kidney morphology in GN rats (n = 10).

By observing the changes in the kidney physiological structure in the GN rat model, we found that the kidney in the control group was bright red with a smooth surface and normal renal tissue shape. In contrast to the control group, kidneys in the model group appeared enlarged with uneven surfaces, exhibiting numerous white spots. However, rats administered TFS capsule, febuxostat, and DTX displayed comparatively smoother kidney surfaces, characterized by a bright red hue and a reduction in white spot areas (Figure 5).

### 4.5 DTX increased kidney index, body weight, and kidney weight

**FIGURE 6:**
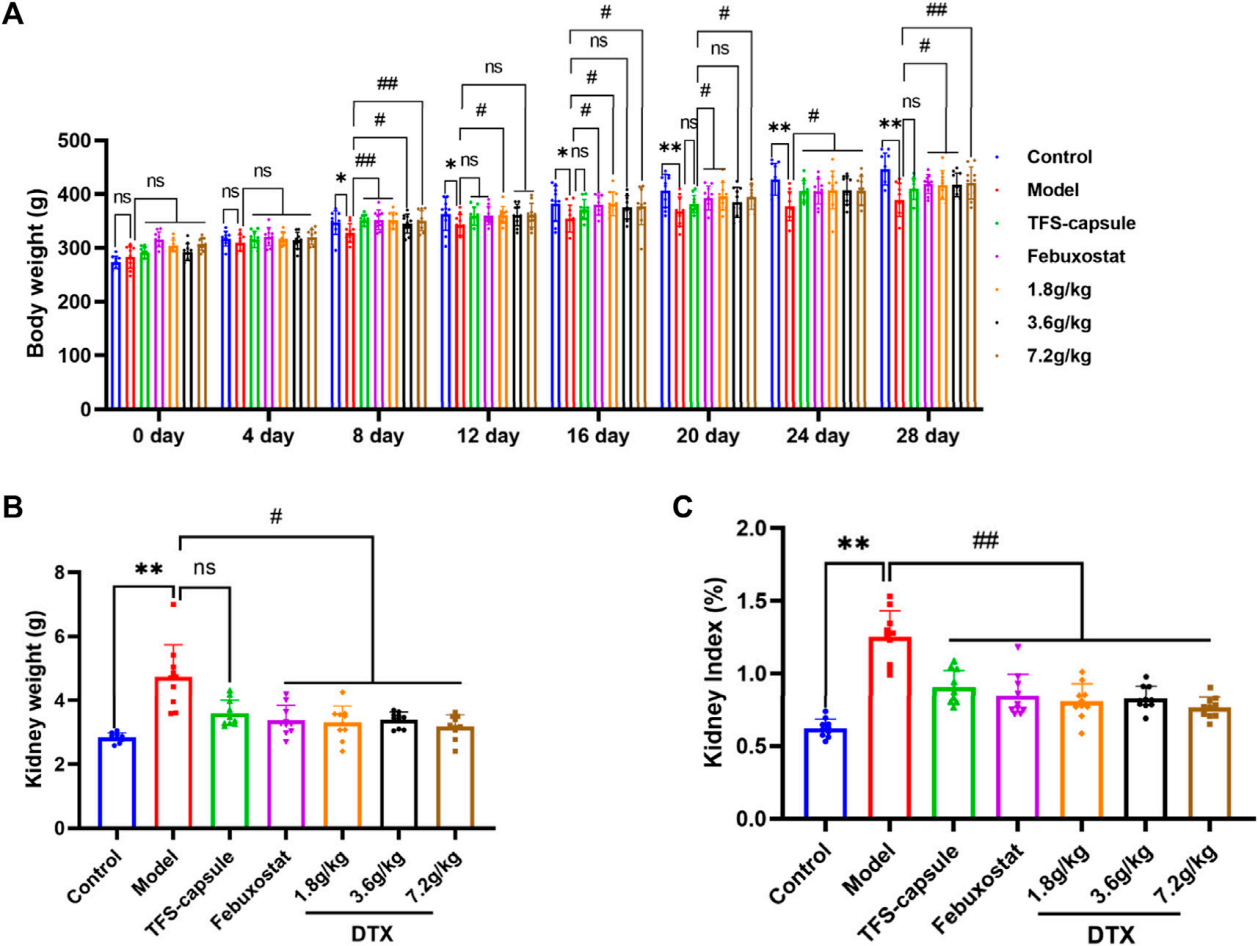
DTX improved kidney morphology in GN rats (n = 10). **(A)** Rat body weight on days 0, 4, 8, 12, 16, 20, 24, and 28; **(B)** rat kidney weight at 24 h after the last administration; **(C)** rat kidney index at 24 h after the last administration. Comparing the reference set as the control group; *p < 0.05 and **p < 0.01. Comparing the reference set as the model group, #*p* < 0.05; ##*p* < 0.01; ns: *p* > 0.05.

We investigated DTX impacts on GN by assessing the rat’s kidney index, body weight, and kidney weight. In comparison to the control group, the model group presented a notable decrease in body weight on days 8, 12, 16, 20, 24, and 28 (*p* < 0.05 or *p* < 0.01) (Figure 6A). In contrast, the DTX 1.8 g/kg group displayed a significant increase in body weight on days 8, 12, 16, 20, 24, and 28 in comparison to the model group (*p* < 0.05 or *p* < 0.01) (Figure 6A). The DTX 3.6 g/kg cohort exhibited a notable increase in body weight on days 8, 24, and 28 (*p* < 0.05) (Figure 6A) while showing an upward trend on days 12, 16, and 20 (*p* > 0.05) (Figure 6C). Moreover, the DTX 7.2 g/kg group demonstrated a significant increase in body weight on days 8, 16, 20, 24, and 28 (*p* < 0.05 or *p* < 0.01) (Figure 6A).

The changes in GN rat status induced by DTX can be preliminarily assessed through renal weight and renal index. Compared to the control group, both kidney index and kidney weight in the model group exhibited significant increases with statistical significance (*p* < 0.01) (Figures 6B, C). Conversely, in comparison to the model group, both kidney index and kidney weight in the DTX groups (1.8, 3.6, and 7.2 g/kg) showed significant decreases with statistical significance (*p* < 0.01 or *p* < 0.05) (Figures 6B, C). These findings suggest that DTX may safeguard the kidneys of rats by enhancing body weight, renal weight, and renal index.

### 4.6 DTX improved UA, Bun, 24-h urinary protein, and allantoin in rats’ urine

Urine UA, Bun, and 24 h urinary protein serve as important indicators for renal function (Kim et al., 2023). Uricase exists in most animals, facilitating uric acid breakdown into allantoin, which is then eliminated from the body (Schlesinger et al., 2023a). Reduced levels of UA, Bun, and allantoin in urine, along with the heightened levels of 24-h urinary protein, typically signify organic kidney function impairment. To explore DTX’s potential in enhancing kidney excretory function, we assessed UA, Bun, 24-h urinary protein, and allantoin in rats’ urine.

**FIGURE 7:**
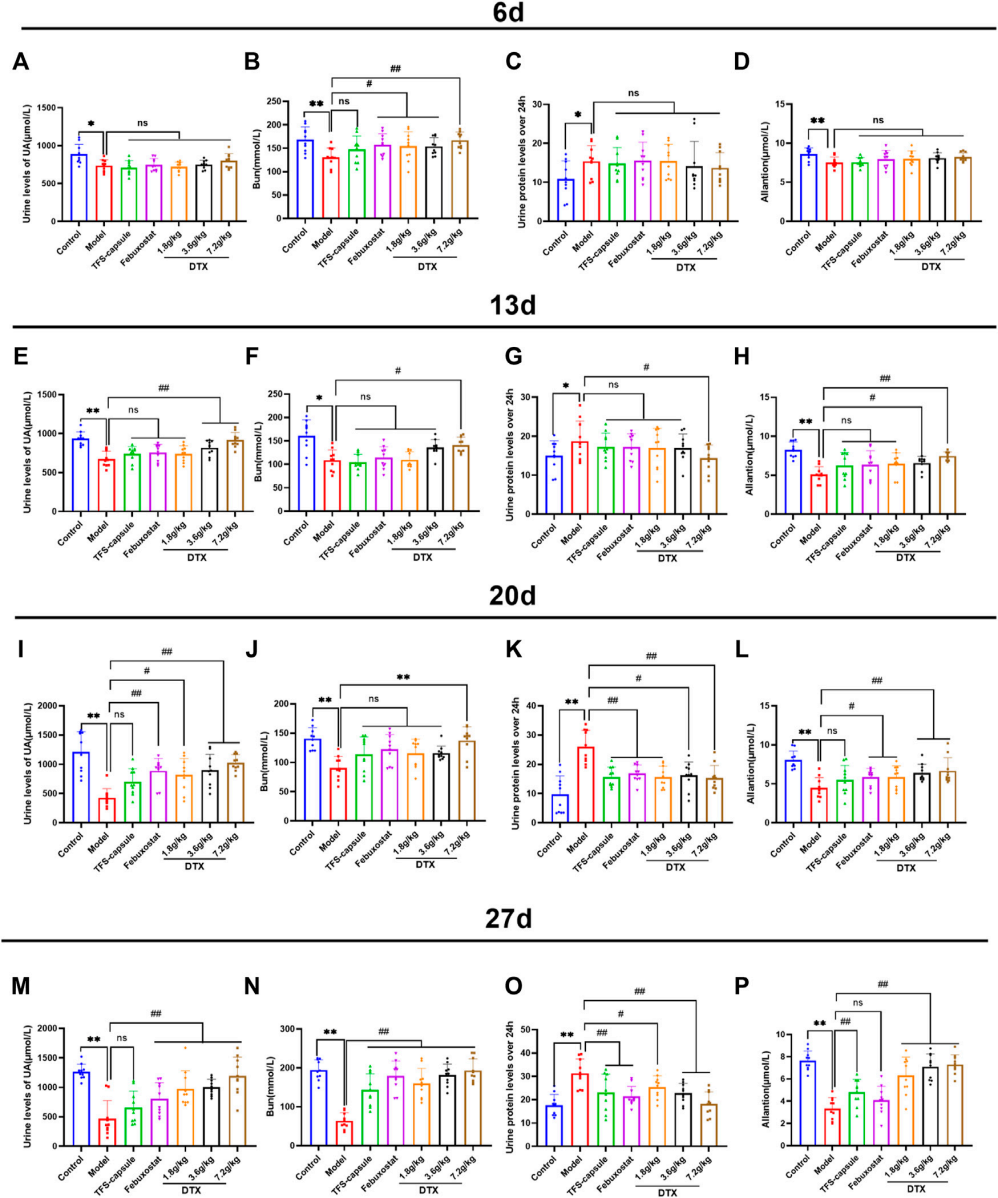
DTX elevated UA, Bun, 24-h urinary protein, and allantoin in rat urine (n = 10). **(A)** Urine UA level of rats on the 6th day, **(B)** urine Bun level of rats on the 6th day, **(C)** 24-h urine protein level of rats on the 6th day, and **(D)** urine allantoin level of rats on the 6th day. **(E)** Urine UA level of the rat on 13th day, **(F)** urine Bun level of the rat on 13th day, **(G)** urine protein level of the rat on 13th day for 24 h, and **(H)** urine allantoin level of the rat on 13th day. **(I)** Urine UA level of rats on the 20th day, **(J)** urine Bun level of rats on the 20th day, **(K)** urine protein level of rats on the 20th day, and **(L)** urine allantoin level of rats on the 20th day. **(M)** urine UA level of rats on the 27th day, **(N)** urine Bun level of rats on the 27th day, **(O)** urine protein level of rats on the 27th day, and **(P)** urine allantoin level of rats on the 27th day. Comparing the reference set as the control group; *p < 0.05 and **p < 0.01. Comparing the reference set as the model group; #*p* < 0.05 and ##*p* < 0.01; ns: *p* > 0.05.

On day 6, we observed a notable reduction in UA, Bun, and allantoin of the model group, while 24-h urinary protein showed a significant increase compared to the control, indicating renal impairment (*p* < 0.05 or *p* < 0.01) (Figures 7A–D). Conversely, compared to the model group, DTX 1.8, 3.6, and 7.2 g/kg groups demonstrated significantly elevated Bun (*p* < 0.05 or *p* < 0.01), with Bun levels in DTX 1.8 and 3.6 g/kg groups showing significant increases (*p* < 0.05) (Figure 7B).

On day 13, experimental findings revealed that in comparison to the control group, the model group exhibited significantly reduced UA, Bun, and allantoin, along with a notable increase in 24-h urinary protein (*p* < 0.05 or *p* < 0.01). Conversely, when compared to the model group, the DTX 7.2 g/kg group displayed significantly elevated UA, Bun, and allantoin (*p* < 0.05 or *p* < 0.01). The DTX 3.6 g/kg group presented notable increases in UA and allantoin levels, along with a notable decrease in 24-h urinary protein (*p* < 0.05 or *p* < 0.01). In the DTX 1.8 g/kg group, there were decreases in UA, Bun, and allantoin levels although not statistically significant (*p* > 0.05). There was a trend toward increased Bun in the DTX 3.6 g/kg group (*p* > 0.05). The urinary protein of rats in DTX 1.8 and 3.6 g/kg groups decreased at 24 h (*p* > 0.05) (Figures 7E–H).

On day 20, in comparison to the control group, the model group presented notable decreases in UA, Bun, and allantoin, while there was a notable increase in 24-h urinary protein (*p* < 0.01). In comparison to the model group, DTX at 1.8, 3.5, and 7.2 g/kg led to significant increases in UA and allantoin levels (*p* < 0.05 or *p* < 0.01). Additionally, Bun and 24-h urinary protein were significantly decreased in the DTX 7.2 g/kg group (*p* < 0.05 or *p* < 0.01). A notable increase in Bun levels was found in the DTX 7.2 g/kg group (*p* < 0.01), while trends toward increased Bun levels were found in the DTX 1.8 and DTX 3.6 g/kg groups (*p* > 0.05) (Figures 7I–L).

On day 27, in comparison to the control group, significant decreases were found in the model group of UA, Bun, and allantoin levels, while there was a significant increase in 24-h urinary protein (*p* < 0.01). Setting the model group as a reference, DTX at 1.8, 3.6, and 7.2 g/kg led to significant increases in UA, Bun, and allantoin levels (*p* < 0.01), and a significant decrease was found in 24-h urinary protein (*p* < 0.05 or *p* < 0.01) (Figures 7M–P).

### 4.7 DTX decreased serum UA, Bun, Cre, CysC, SAA, α1-MG, and β2-MG in rats

**FIGURE 8:**
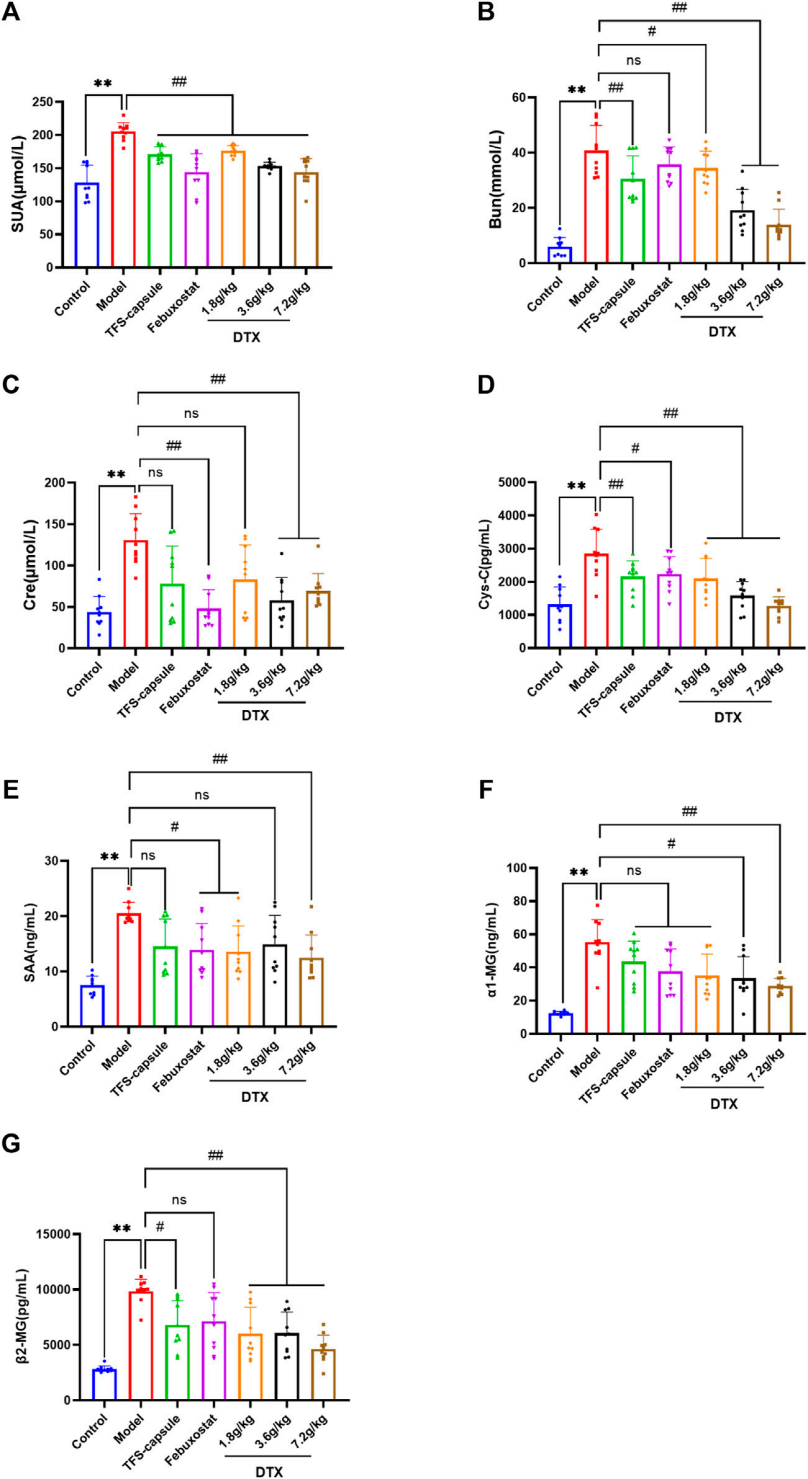
Effects of DTX on serum levels of UA, Bun, Cre, CysC, SAA, α1-MG, and β2-MG in GN rats (n = 10). **(A)** Serum UA levels. **(B)** Serum Bun level. **(C)** Serum Cre level. **(D)** Serum CysC level. **(E)** Serum SAA level. **(F)** Serum α1-MG level. **(G)** Serum β2-MG level. Comparing the reference set as the control group; *p < 0.05 and **p < 0.01. Comparing the reference set as the model group; #*p* < 0.05 and ##*p* < 0.01; ns: *p* > 0.05.

Through urinary indicator analysis in rats, we observed a significant enhancement in renal excretory function following DTX administration in GN rats. To further verify the improving effect of DTX on GN, we assessed serum levels of UA, Bun, Cre, CysC, SAA, α1-MG, and β2-MG. Compared with the control group, the model group exhibited a substantial increase in serum UA, Cre, Bun, CysC, SAA, α1-MG, and β2-MG, with statistical significance (*p* < 0.01). Conversely, in the DTX 7.2 g/kg group, there was a significant reduction in UA, Bun, Cre, Cys-C, SAA, α1-MG, and β2-MG (*p* < 0.01). Decreasing indicators in the DTX 3.6 g/kg group included UA, Bun, Cre, Cys-C, α1-MG, and β2-MG (*p* < 0.05 or *p* < 0.01), while in the DTX 1.8 g/kg group, it included UA, Bun, Cys-C, SAA, and β2-MG (*p* < 0.05 or *p* < 0.01). Although SAA in the DTX 3.6 g/kg group and Cre and α1-MG in the DTX 1.8 g/kg group had decreasing trends, the differences were not statistically significant compared to the model group (*p* > 0.05) (Figures 8A–G).

### 4.8 DTX downregulates the levels of XOD, Hyp, α-SMA, and COL-Ⅳ in rat renal tissue

**FIGURE 9:**
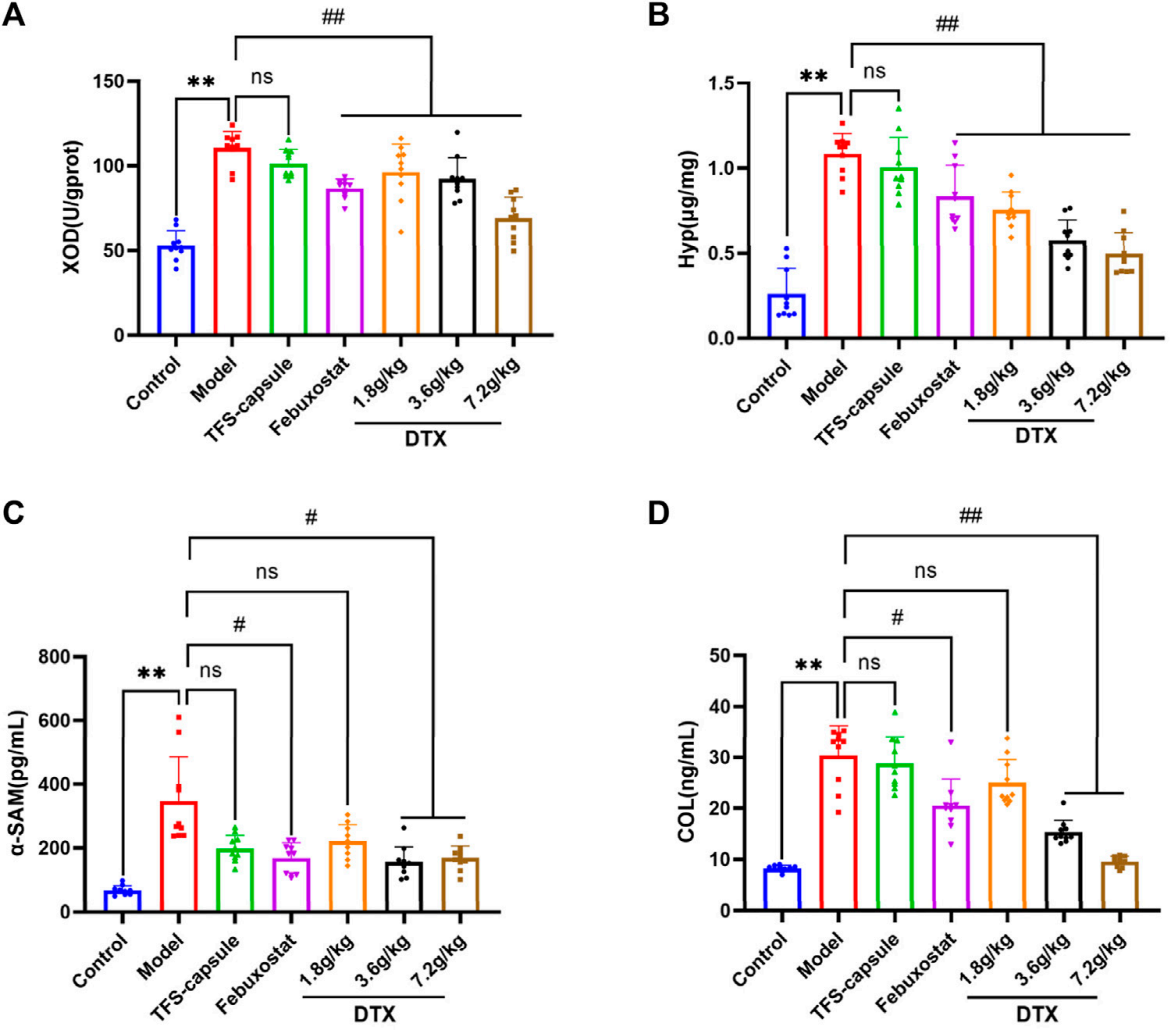
DTX reduced XOD, Hyp, α-SMA, and COL-Ⅳ levels in kidney tissues of GN rats (n = 10). **(A)** XOD in kidney tissue. **(B)** Hyp in kidney tissue. **(C)** α-SMA in kidney tissue. **(D)** COL-Ⅳ in kidney tissue. Comparing the reference set as the control group; **p < 0.01. Comparing the reference set as the model group; #*p* < 0.05 and ##*p* < 0.01; ns: *p* > 0.05.

Compared to the control group, renal tissue XOD, Hyp, α-SMA, and COL-Ⅳ levels were notably increased within the model group, indicating renal impairment (*p* < 0.01) (Figures 9A–D). Conversely, DTX treatment at 3.6 and 7.2 g/kg as well as 1.8 g/kg led to a substantial reduction in XOD, Hyp, α-SMA, and COL-Ⅳ levels in comparison to the model group, demonstrating significant improvements (*p* < 0.05 or *p* < 0.01) (Figures 9A–D). Although α-SMA and COL-Ⅳ in the DTX 1.8 g/kg group showed a decreasing trend, changes were not statistically significant compared to the model group (*p* > 0.05) (Figures 9C, D).

### 4.9 DTX ameliorated the inflammatory infiltration of GN rat kidney tissue

**FIGURE 10:**
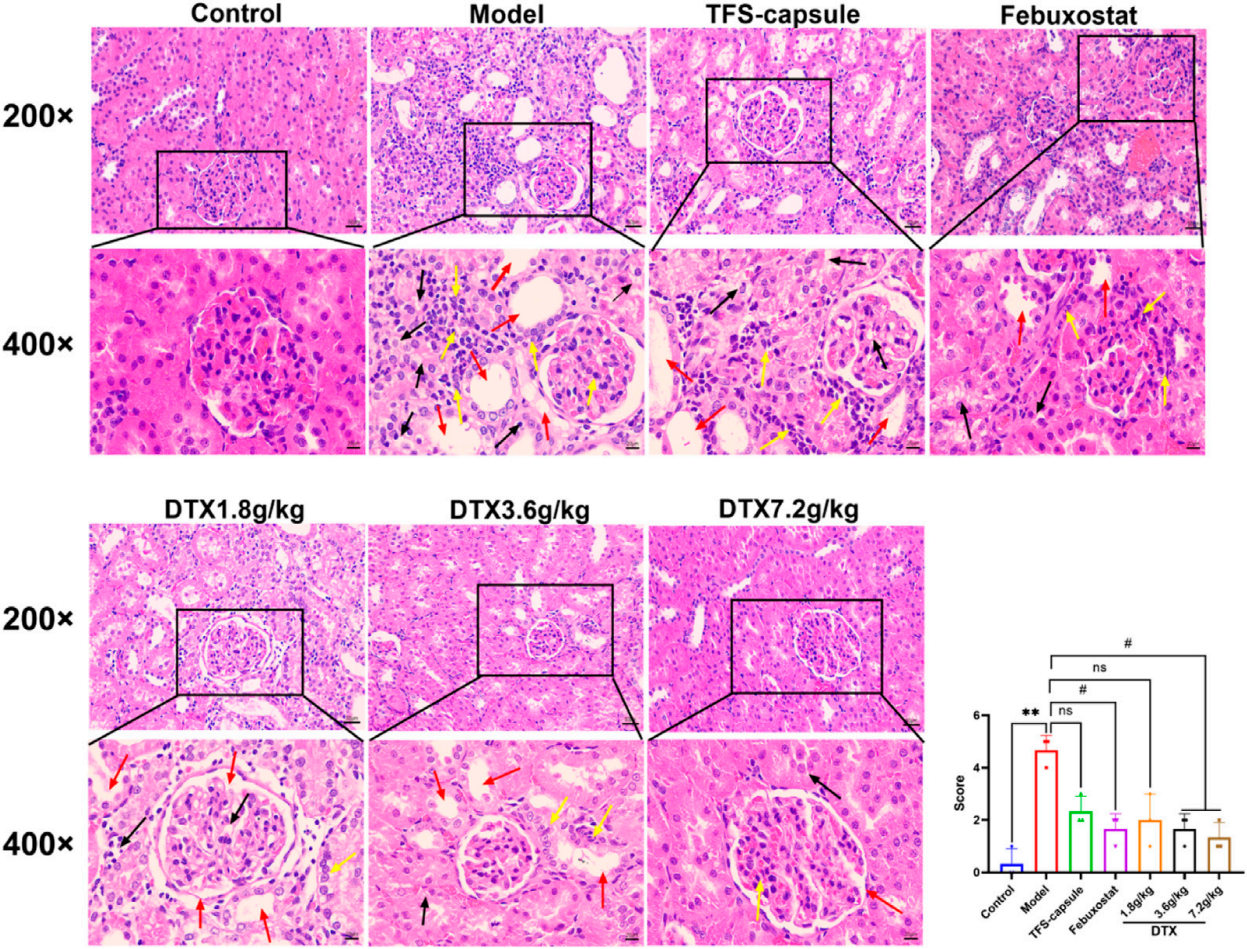
DTX suppressed inflammatory infiltration of kidney tissues in GN rats. H&E staining of rat kidneys (200× and 400×) (n = 3). Red arrow, renal tubule lumen dilation; black arrow, degeneration of renal tubular epithelial cells; yellow arrow, inflammatory infiltration. Comparing the reference set as the control group; **p < 0.01. Comparing the reference set as the model group; #*p* < 0.05; ns: *p* > 0.05.

Renal inflammation and changes in renal cell morphology were assessed using H&E staining. In the control group, kidney morphology appeared normal, with no mesangial cell proliferation. The renal tubular epithelial cells were well-arranged with clear outlines, and there were no evident signs of degeneration, luminal expansion, inflammatory cell infiltration, or apparent pathological changes. In contrast, the model group exhibited pronounced renal tissue abnormalities characterized by extensive edema of tubular epithelial cells, cytoplasmic vacuolation, severe luminal dilation, and substantial inflammatory cell infiltration in the interstitium. However, DTX-treated groups (1.8, 3.6, and 7.2 g/kg) displayed reduced inflammatory cell infiltration in the renal interstitium, limited renal tubule dilation, absence of renal tubular epithelial cell degeneration, and overall injury severity mitigation compared to the model group (Figure 10). These findings indicate that DTX may shield the kidneys by mitigating inflammatory infiltration and bolstering renal structure.

### 4.10 DTX reduced collagen fiber deposition in the GN rat kidney

**FIGURE 11:**
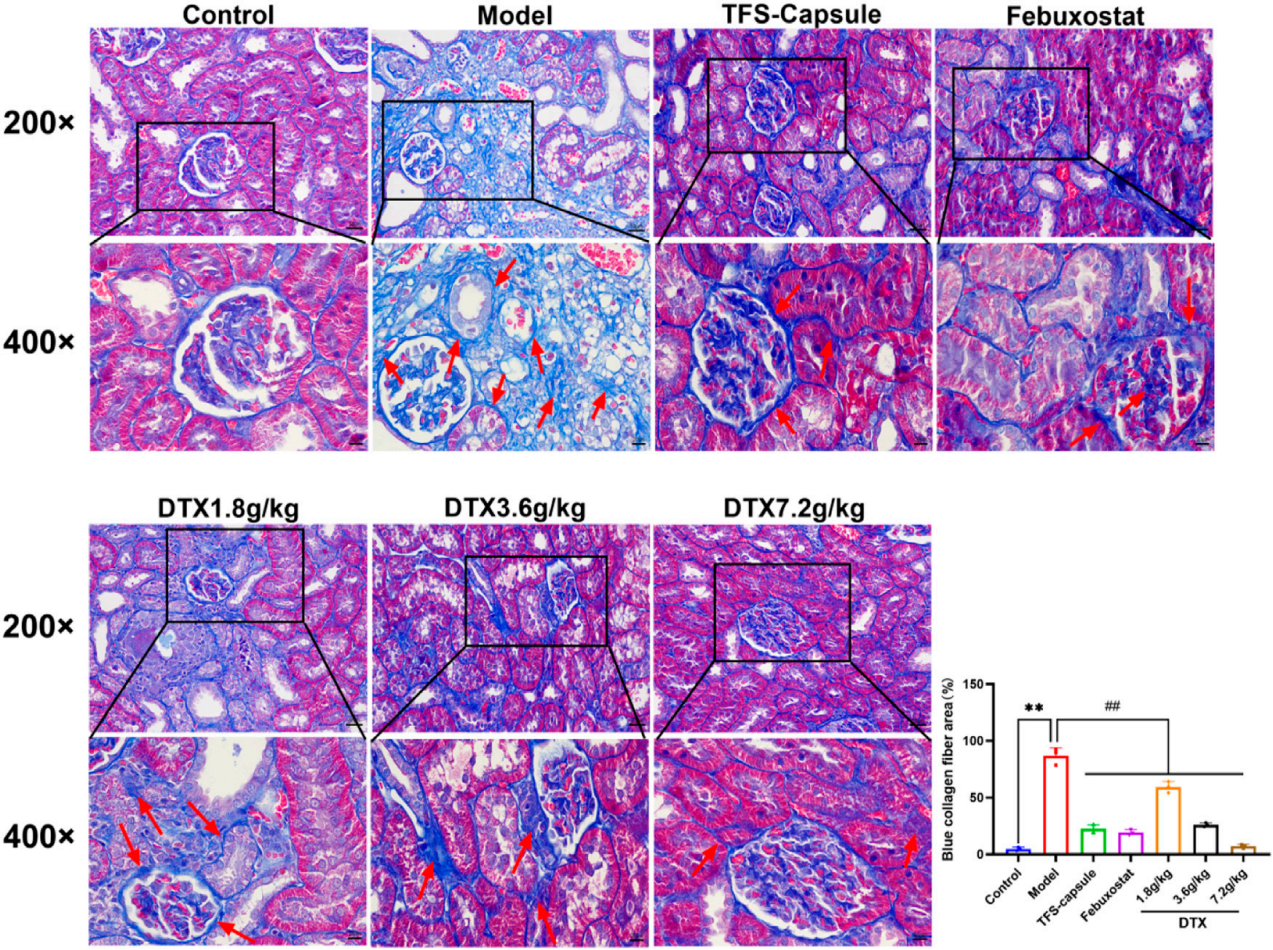
DTX improved collagen fiber deposition in the GN rat kidney (200× and 400×) (n = 3). Comparing the reference set as the control group; **p < 0.01. Comparing the reference set as the model group; ##*p* < 0.01. Red arrow, collagen fibrosis.

Masson staining revealed increased collagen fiber deposition in rats’ kidneys in the model group in comparison to normal control, with statistical significance (*p* < 0.01) (Figure 11). Conversely, in DTX 1.8, 3.6, and 7.2 g/kg groups, collagen fiber deposition in renal tissue was notably reduced in comparison to the model group, with statistical significance (*p* < 0.01) (Figure 11). These findings indicate that DTX may shield kidneys by mitigating collagen fiber deposition.

### 4.11 DTX reduced the thickness of the basement membrane in renal tissues of GN rats

**FIGURE 12:**
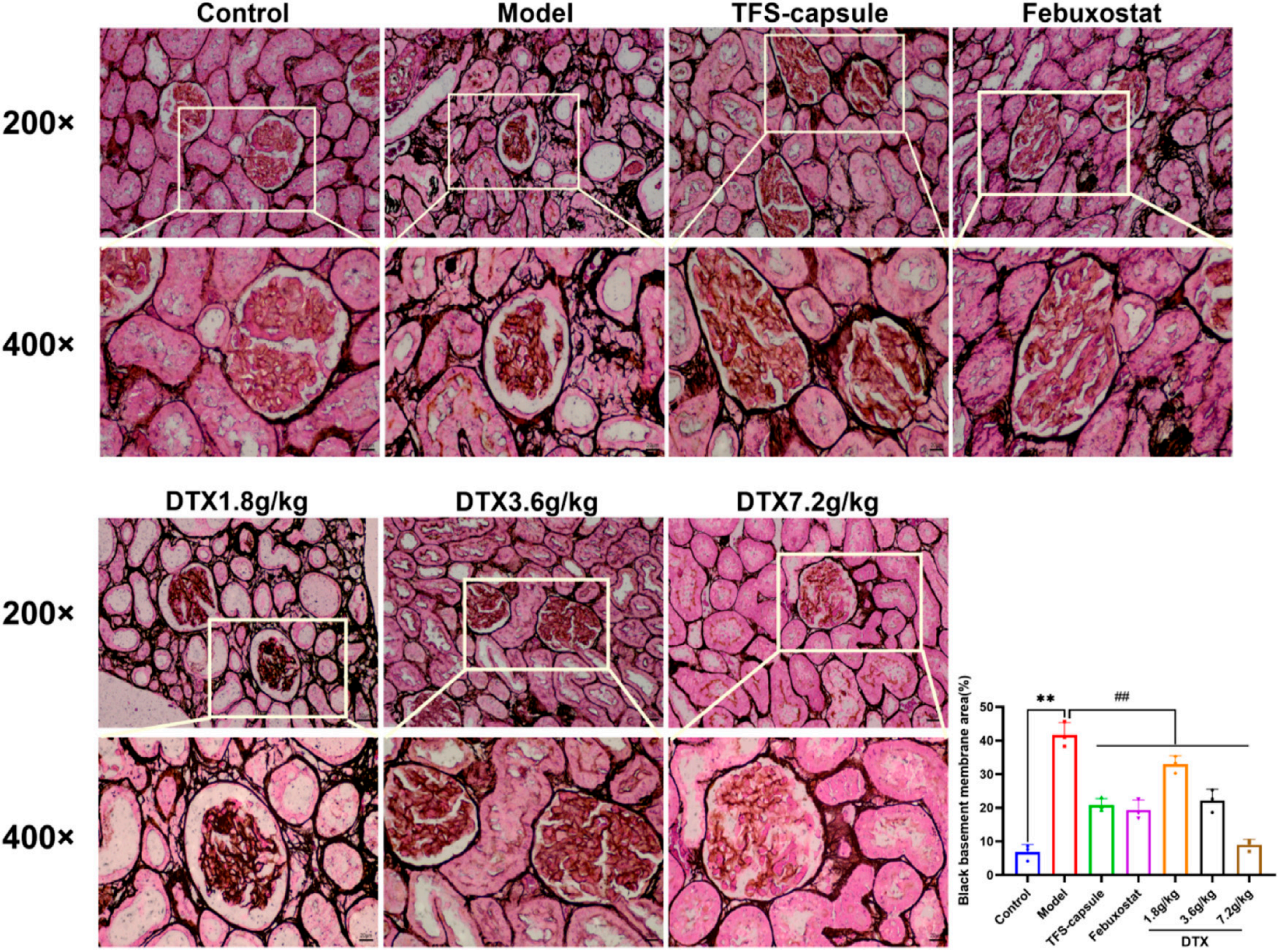
DTX reduced basement membrane thickness in kidney tissues of GN rats (200× and 400×) (n = 3). Comparing the reference set as the control group; **p < 0.01. Comparing the reference set as the model group; ##*p* < 0.01.

PASM staining revealed glomerular atrophy and basement membrane thickening. Compared to the control group, the model group presented observable thickening of the basement membrane and numerous instances of glomerular atrophy and sclerosis (*p* < 0.01) (Figure 12). However, in DTX 1.8, 3.6, and 7.2 g/kg groups, no notable tubular atrophy or thickening of the basement membrane was observed. Additionally, glomerular atrophy and sclerosis degrees were markedly improved compared to the model group (*p* < 0.01) (Figure 12). The outcomes indicated that DTX may protect kidneys by reducing basement membrane thickness and ameliorating glomerular atrophy.

### 4.12 DTX reduced the degree of urate deposition in the renal tissues of GN rats

**FIGURE 13:**
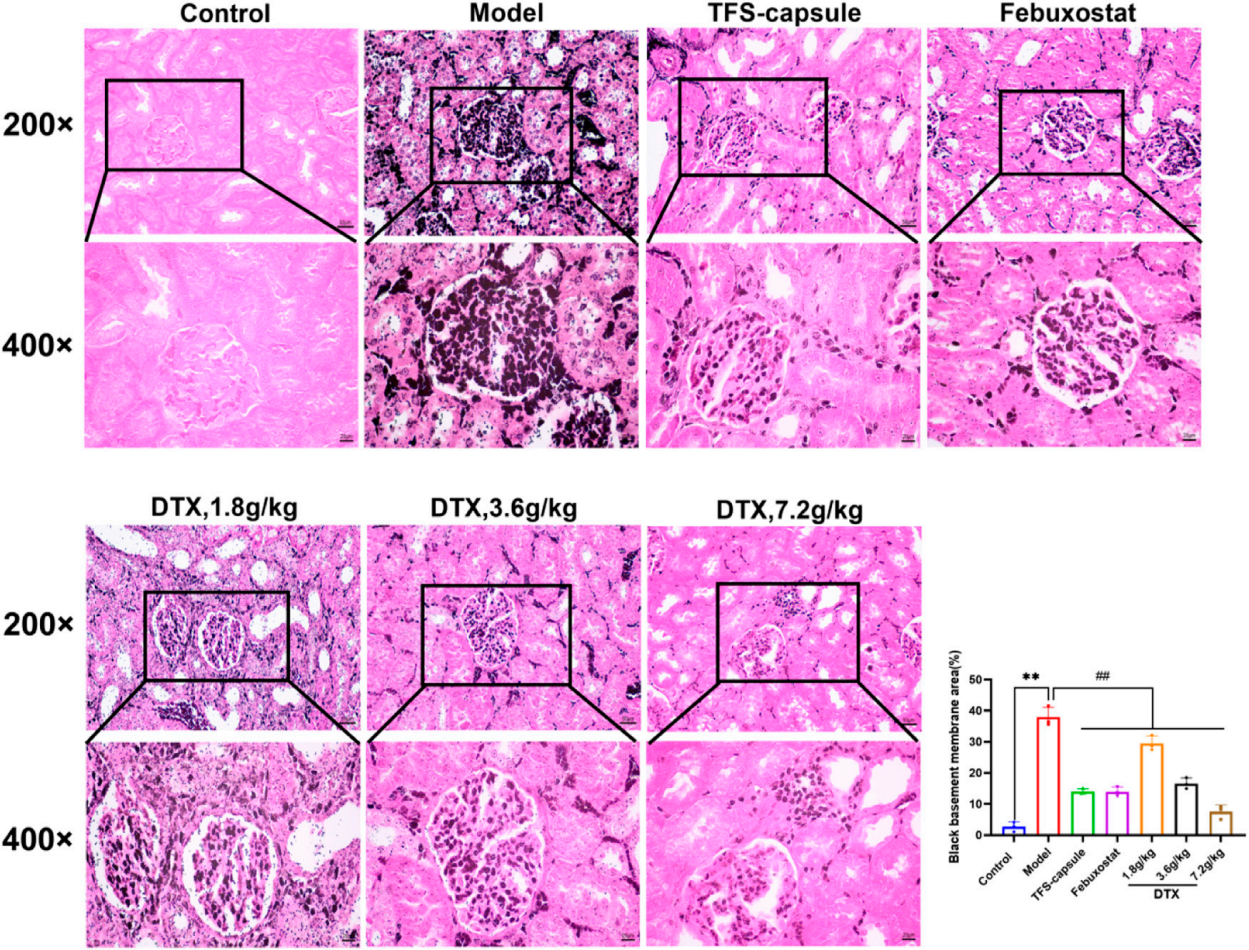
DTX reduced urate deposition in kidney tissues of GN rats. Gomori staining of rat kidneys (200× and 400×) (n = 3). Comparing the reference set as the control group; **p < 0.01. Comparing the reference set as the model group; ##*p* < 0.01.

Gomori staining was used to observe urate deposition. The model group exhibited severe urate deposition compared to the control group. However, in DTX 1.8, 3.6, and 7.2 g/kg groups, urate deposition decreased progressively compared to the model group (Figure 13). These findings imply that DTX may safeguard the kidneys by reducing urate deposition.

### 4.13 Impact of DTX on serum CRP, IL-18, TGF-β1, IL-1β, and TNF-α in rats

**FIGURE 14:**
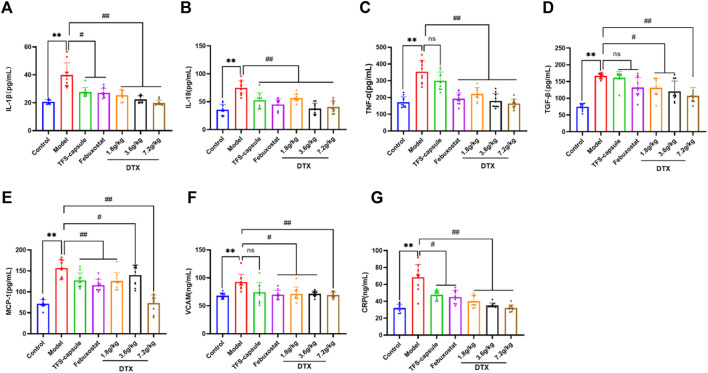
DTX alleviated inflammatory factors release in the GN rat serum (n = 10); **(A)** IL-1β in the rat serum. **(B)** IL-18 in the rat serum. **(C)** TNF-α in the rat serum. **(D)** TGF-β1 in the rat serum. **(E)** MCP-1 in the rat serum. **(F)** VCAM in the rat serum. **(G)** CRP in the rat serum. Comparing the reference set as the control group, **p < 0.01. Comparing the reference set as the model group; #*p* < 0.05 and ##*p* < 0.01; ns: *p* > 0.05.

To evaluate the potential anti-inflammatory effects of DTX, serum CRP, IL-18, TGF-β1, IL-1β, and TNF-α in rats were measured. Compared to normal control, the model group presented significantly elevated serum CRP, IL-18, TGF-β1, IL-1β, and TNF-α in rats (*p* < 0.01) (Figures 14A–D, G). Conversely, DTX treatment at 1.8, 3.6, and 7.2 g/kg resulted in a notable reduction in serum CRP, IL-18, TGF-β1, IL-1β, and TNF-α when compared to the model cohort (*p* < 0.05 or *p* < 0.01) (Figures 14A–D, G). The outcomes indicate that DTX exhibits anti-inflammatory properties and may protect kidney function.

### 4.14 DTX decreased the serum MCP-1 level in rats

To explore whether DTX can mitigate chemokine MCP-1 release, which plays a role in inducing renal tubule atrophy and interstitial fibrosis, thereby hastening GN progression, we measured the serum MCP-1 levels in rats. The serum MCP-1 content in the model group was markedly elevated compared to the control group (*p* < 0.01). However, in comparison to the model group, serum MCP-1 levels in the DTX 1.8, 3.6, and 7.2 g/kg groups exhibited significant decreases (*p* < 0.05 or *p* < 0.01) (Figure 14E).

### 4.15 DTX decreased the content of VCAM-1 in rat serum

VCAM-1 belongs to the immunoglobulin superfamily of adhesion molecules and is involved in inflammatory responses and immune regulation. Upregulated VCAM-1 expression prompts enhanced infiltration of mononuclear macrophages in kidney tissue and their adhesion to endothelial cells, resulting in increased inflammatory cells, aggravated inflammatory response, extracellular matrix deposition, and ultimately kidney injury. VCAM-1 in the rat serum was determined. Compared with the control group, serum VCAM-1 in the model group was significantly increased (*p* < 0.01). Compared with the model group, serum VCAM-1 content in DTX 1.8, 3.6, and 7.2 g/kg groups was significantly decreased (*p* < 0.05 or *p* < 0.01) (Figure 14F).

### 4.16 DTX suppressed the TLR4/MyD88/NF-κB pathway-related protein expression

**FIGURE 15:**
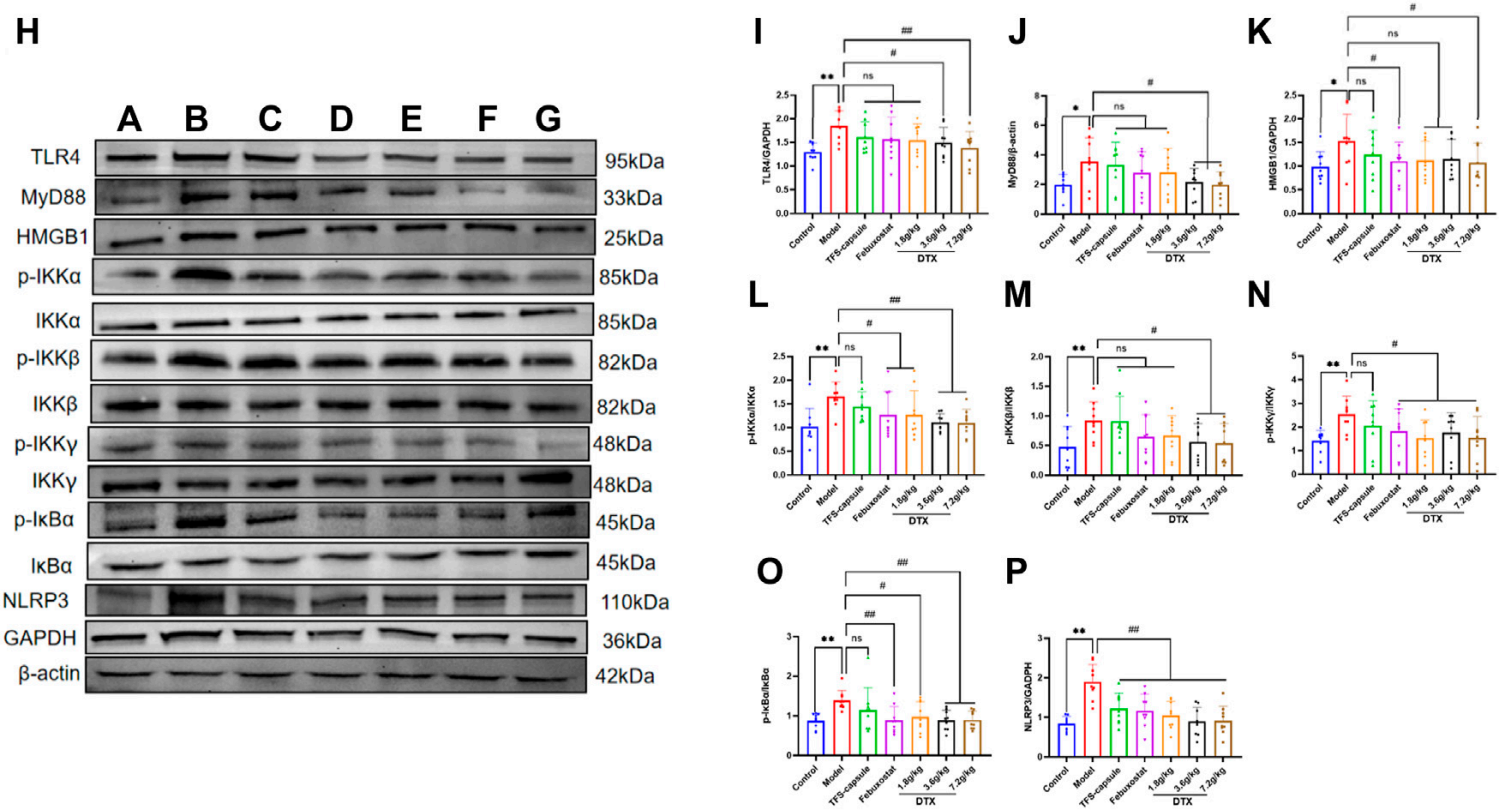
DTX modulated protein expression in GN rat kidney tissues (n = 3). **(A)** control group, **(B)** model group, **(C)** TFS capsule group, **(D)** febuxostat group, **(E)** DTX 1.8 g/kg dose group, **(F)** DTX 3.6 g/kg dose group, **(G)** DTX 7.2 g/kg dose group, and **(G)** DTX 7.2 g/kg dose group. **(H)** Protein plotting of TLR4, MyD88, HMGB1, p-IKKα/IKKα, p-IKKβ/IKKβ, p-IKKγ/IKKγ, p-IκBα/Iκbα, and NLRP3. **(I)** TLR4 protein expression. **(J)** MyD88 protein expression. **(K)** HMGB1 protein expression. **(L)** p-IKKα/IKKα protein expression. **(M)** p-IKKβ/IKKβ protein expression. **(N)** p-IKKγ/IKKγ protein expression. **(O)** p-IκBα/IκBα protein expression. **(P)** NLRP3 protein expression. Comparing the reference set as the normal group; *p < 0.05. Comparing the reference set as the model group; #*p* < 0.05 and ##*p* < 0.01; ns: *p* > 0.05.

The potential anti-GN mechanism of DTX may involve the modulation of proteins within the TLR4/MyD88/NF-κB pathway. Therefore, proteins TLR4, MyD88, HMGB1, p-IKKα/IKKα, p-IKKβ/IKKβ, p-IKKγ/IKKγ, p-IκBα/IκBα, and NLRP3 in rat renal tissue were analyzed by Western blotting (Figures 15A–G). Compared to the control group, the model group presented notable upregulation in protein expressions in TLR4, MyD88, HMGB1, p-IKKα/IKKα, p-IKKβ/IKKβ, p-IKKγ/IKKγ, p-IκBα/IκBα, and NLRP3 (*p* < 0.05 or *p* < 0.01) (Figures 15H–P). Conversely, compared with the model group, the DTX 1.8 g/kg group exhibited significant downregulation in p-IKKα/IKKα, p-IKKγ/IKKγ, p-IκBα/IκBα, and NLRP3 (*p* < 0.05 or *p* < 0.01) (Figures 15H, L, N, O). Moreover, DTX 3.6 and 7.2 g/kg groups demonstrated significant downregulation in protein expressions of TLR4, MyD88, HMGB1, p-IKKα/IKKα, p-IKKβ/IKKβ, p-IKKγ/IKKγ, p-IκBα/IκBα, and NLRP3 (*p* < 0.05 or *p* < 0.01) (Figures 15H, J, L–P). The DTX 7.2 g/kg group significantly downregulated HMGB1 protein expression (*p* < 0.05 or *p* < 0.01) (Figures 15H, K). Furthermore, MyD88, HMGB1, and p-IKKβ/IKKβ in the DTX 1.8 g/kg group and HMGB1 in the 3.6 g/kg group exhibited downward trends without statistical significance (*p* > 0.05) (Figures 15J, K, M).

### 4.17 DTX decreased NF-κB in renal tissues of GN rats

**FIGURE 16:**
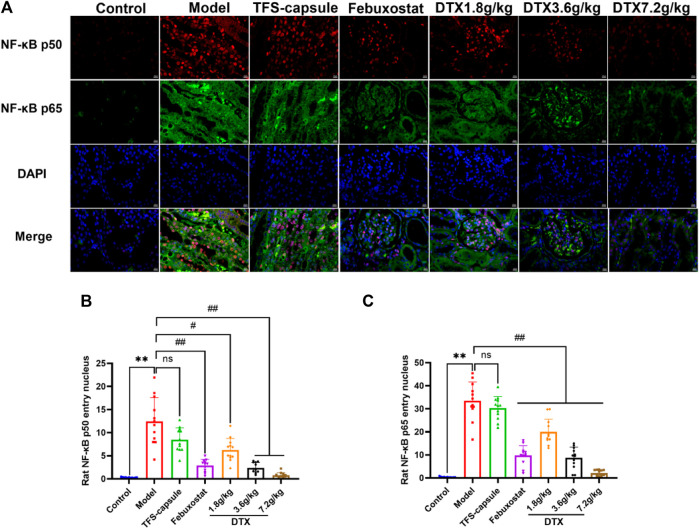
DTX reduced NF-κB expression in renal tissues of GN rats. **(A)** Rat kidney tissue (n = 3) immunofluorescence map. **(B)** Immunofluorescence surface density of the rat kidney tissue of NF-κB p50. **(C)** Immunofluorescence surface density of the rat kidney tissue of NF-κB p65. Red, NF-κB p50; green, NF-κB p65; and blue, 4, 6-diamino-2-styrenol (DAPI). Comparing the reference set as the control group; **p < 0.01; Comparing the reference set as the model group; #*p* < 0.05 and ##*p* < 0.01. ns showed no statistical significance.

The NF-κB dimer plays as a mediator in the TLR4/MyD88/NF-κB pathway, contributing significantly to GN genesis. Thus, we examined the NF-κB dimer protein expression in the rat renal tissue nucleus using an immunofluorescence method. In our findings, the nucleus appeared blue under ultraviolet excitation, while NF-κB p50 was visualized in red and NF-κB p65 in green. Compared to the control group, NF-κB p50 and NF-κB p65 expressions in renal tissue nucleus were markedly elevated in the model group, demonstrating statistical significance (*p* < 0.01) (Figures 16A–C). However, in comparison to the model group, NF-κB p50 and NF-κB p65 in the renal cell nucleus of rats treated with DTX at 1.8, 3.6, and 7.2 g/kg were notably reduced (*p* < 0.05 or *p* < 0.01) (Figures 16A–C). These findings indicate that DTX may safeguard kidney function by suppressing the NF-κB dimer.

### 4.18 Molecular docking analysis

**FIGURE 17:**
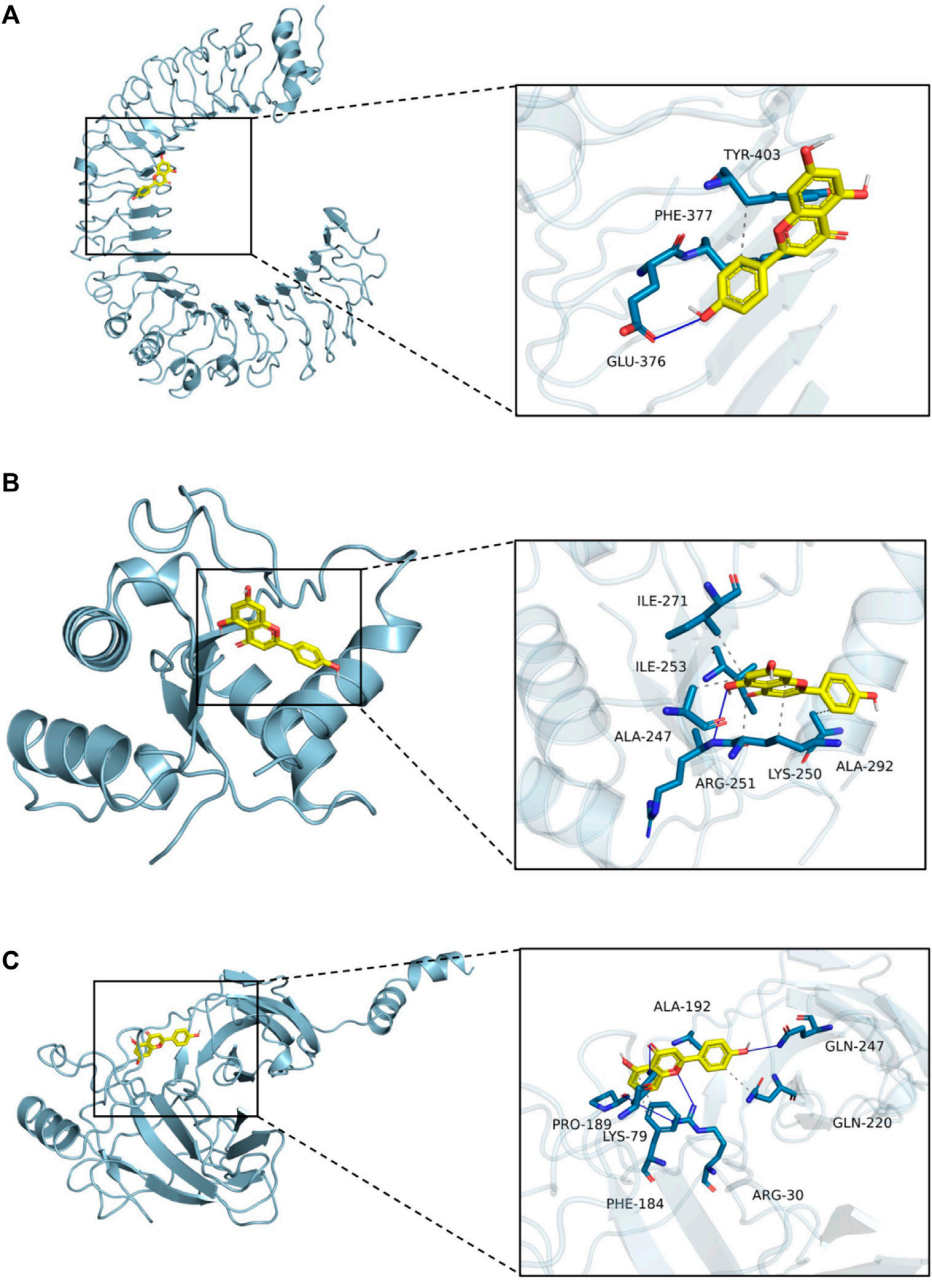
Molecular docking. **(A)** Molecular docking mode of apigenin and TLR4. **(B)** Molecular docking mode of apigenin and MyD88. **(C)** Molecular docking mode of apigenin and NF-κB.

Apigenin, a flavonoid, promotes uric acid excretion and improves renal function. In this experiment, apigenin was found to be the main active component in DTX via UHPLC-OE-MS analysis. Therefore, this study utilized molecular docking to further evaluate the affinity between apigenin and TLR4, MyD88, and NF-κB. The results showed that the binding energies of apigenin with TLR4, MyD88, and NF-κB were −6.2 kcal/mol, −8.0 kcal/mol, and −6.1 kcal/mol, respectively, and had good affinity (Table 4; Figure 17).

**TABLE 4:** Molecular docking results.

Compound	Protein	Combining energy
Apigenin	TLR4	−6.2 kcal/mol
	MyD88	−8.0 kcal/mol
	NF-κB	−6.1 kcal/mol

## 5 Discussion

GN arises from excessive production or reduced excretion of UA in the body. This condition leads to a prolonged state of blood UA saturation, resulting in the deposition of uric acid crystals within the renal medulla, interstitium, and distal collecting ducts of the kidney. Over time, this deposition contributes to substantial renal lesions, including edema, urinary calculi, elevated urinary UA, and functional damage of renal tubules (Yanai et al., 2021; Shi et al., 2023). Dai medicine embodies the collective wisdom accumulated by the Dai people in China through generations of battling diseases. It amalgamates insights from ancient Indian medicine and traditional Chinese medicine, bearing distinct national and regional features (TAN et al., 2013). Among the repertoire of remedies in Dai medicine, DTX stands out as a widely employed compound for managing gout. Renowned for its potent efficacy and minimal side effects, DTX has garnered recognition (Qinghua et al., 2014a; Qinghua et al., 2014b; Feng, 2015). Some studies have shown that DTX is rich in flavonoids and volatile constituents (Feifan et al., 2023). Among them, flavonoid components inhibit the expression of TLR4, MyD88, NLRP3, and other proteins by acting on the TLR/MyD88/NLRP3 and other related protein pathways, resulting in a reduction in the release of inflammatory factors, thus producing an anti-inflammatory effect in order to inhibit the occurrence and development of gout (Xueyan et al., 2021). Volatile components such as α-pinene may play a role in protecting the kidneys by lowering plasma urea and creatinine (Noroozi et al., 2024).

In our investigation, we observed notable differences in rat kidneys treated with DTX compared to those in the model group. Specifically, DTX-treated rat kidneys appeared smoother on the surface and exhibited a brighter red color, showing reduced areas of white spots. Additionally, we assessed the renal weight and renal index of rats as indicators of changes in their kidney status induced by DTX treatment. In comparison to the model group, both kidney weight and kidney index were significantly decreased in the DTX treatment group. These findings suggest that DTX may safeguard rat kidneys with GN by enhancing kidney morphology and reducing both kidney weight and kidney index.

UA, Cre, Bun, 24-h urinary protein, and allantoin serve as crucial evaluation indexes for assessing renal function. UA is a metabolite of purine in the body, and its accumulation leads to UA deposition in renal tubules, permanent kidney damage in renal interstitium, and finally GN (Yongsheng et al., 2022). Both Bun and Cre are primarily eliminated via glomerular filtration. When the glomerular filtration rate decreases significantly, urinary excretion of Bun and Cre decreases, resulting in elevated concentrations of Bun and Cre in the blood. Bun and Cre are primarily eliminated via glomerular filtration. When the glomerular filtration rate decreases severely, the urinary excretion of Bun and Cre decreases, resulting in elevated concentrations of Bun and Cre in the blood (Zhang et al., 2023b). Extensive proteinuria has been shown to directly harm the kidneys, exacerbating pre-existing glomerular damage and promoting glomerular sclerosis (Elsaid et al., 2024). In mammals other than the human body, urate oxidase converts uric acid into allantoin, which is then excreted from the body, mitigating renal damage caused by uric acid (Mian et al., 2021). The findings revealed notable alterations in urinary and serum parameters among experimental groups. Specifically, in the model group, urinary levels of UA, Bun, and allantoin exhibited significant reductions, whereas urinary protein levels at 24 h significantly increased. Additionally, serum concentrations of UA, Bun, and Cre were notably elevated. Conversely, DTX treatment led to elevated levels of urinary UA, Bun, and allantoin, coupled with decreased levels of urinary protein and serum UA, Bun, and Cre at 24 h. These observations suggest that DTX may mitigate GN by enhancing glomerular filtration function and renal tubular secretion and reabsorption.

In clinical studies, elevated serum levels of CysC, SAA, α1-MG, and β2-MG have been observed in patients with GN, indicating their involvement in GN pathogenesis. CysC serves as an endogenous marker that reliably reflects the glomerular filtration rate and as a marker of early renal function injury (Wang and Wang, 2024). SAA functions as an acute phase reaction protein, exhibiting increased levels in response to renal capillary endothelial injury and abnormal glomerular basement membrane function (Cai et al., 2020). Furthermore, SAA, functioning as an inflammatory mediator, can stimulate neutrophils to release a plethora of inflammatory factors, exacerbating cellular oxidative stress and contributing to the degeneration and necrosis of renal vessels and parenchymal cells (Konstandi et al., 2019). Additionally, α1-MG and β2-MG, low molecular weight proteins, primarily undergo glomerular filtration and subsequent renal tubular reabsorption. Elevated serum levels of α1-MG and β2-MG serve as effective markers for assessing renal tubular function impairment, indicating compromised glomerular filtration and tubular reabsorption functions (Yican et al., 2021). Our experimental findings revealed significantly elevated levels of CysC, SAA, α1-MG, and β2-MG in the model group, whereas DTX administration effectively mitigated these levels, suggesting its potential to ameliorate GN clinical indicators. This reduction in glomerular and renal tubular damage signifies a positive impact on retarding GN progression.

XOD serves as a pivotal enzyme catalyzing the conversion of hypoxanthine to xanthine and further to uric acid (Yican et al., 2021). Elevated XOD activity signifies increased uric acid production, a primary instigator of GN (Wang et al., 2024b). Hyp is one of the characteristic components of collagen, reflecting tissue fibrosis degree. By quantifying the Hyp content in kidney tissue, the breakdown of collagen can be evaluated, providing insights into tissue remodeling processes (Haimei et al., 2022). COL-Ⅳ serves as a pivotal constituent of the extracellular matrix, while α-SMA serves as a marker for interstitial matrix formation. Excessive deposition of both entities can precipitate renal fibrosis and impede renal function (Zhou et al., 2021). The findings revealed notable elevations in XOD, Hyp, α-SMA, and COL-Ⅳ in the renal tissue of rats in the model group. Conversely, renal tissues of rats in the DTX group exhibited reduced levels of XOD, Hyp, α-SMA, and COL-Ⅳ. This suggests that DTX may mitigate renal fibrosis by suppressing uric acid production, thereby exerting a protective effect on kidneys and informing the clinical treatment of GN.

In our experiment, GN rats induced by adenine and potassium oxazinate exhibited morphological and pathophysiological alterations reminiscent of human GN. Our experimental findings confirm the successful replication of the GN model and the efficacy of drug intervention in kidney tissue. According to H&E, Masson, PASM, and Gomori staining, the model group exhibited renal inflammation, structural damage of renal tubular epithelial cells, increased collagen fiber accumulation, basement membrane thickening, massive atrophy, glomeruli sclerosis, and urate massive deposition. Conversely, DTX treatment markedly mitigated renal inflammatory responses, ameliorated the structure of renal tubular epithelial cells, attenuated collagen fiber deposition, and reduced basement membrane thickness. Meanwhile, glomerular atrophy, sclerosis, and urate deposition were also significantly improved. These observations suggested that DTX possesses anti-GN properties by enhancing the pathological state of renal tissue and mitigating urate deposition.

Kidney inflammation is intricately linked with GN (Wang et al., 2024a). IL-1β and IL-18 both belong to the IL-1 cytokine superfamily and are secreted mainly by activated macrophages, renal endothelial cells, and renal tubular epithelial cells. IL-1β prompts immune cell infiltration into damaged tissues, and studies indicate that IL-1β receptor antagonists exhibit promising therapeutic effects on gouty arthritis and GN (Schlesinger et al., 2023b). IL-18 typically resides within cells as inactive precursors, and upon cleavage, mature IL-18 is released into the cell, exerting immune activation effects. It induces the expression of chemokines, pro-inflammatory factors, and adhesion factors, thereby participating in the inflammatory response associated with kidney damage (Liu et al., 2023). TNF-α, originating from macrophages, plays a multifaceted role in the inflammatory response. Research indicates a positive correlation between TNF-α and kidney injury severity (Ye et al., 2023). As a pro-inflammatory cytokine, TNF-α aggravates the inflammatory response of neutrophils and functions as a key factor in gout generation and persistence (Chen J. et al., 2023). TGF-β1 is predominantly expressed in renal tubular epithelial cells, increasing extracellular matrix expression by stimulating myofibroblasts, enhancing adhesion between cells and matrix, and promoting the aggregation of the extracellular matrix in the renal interstitium, which is an important cytokine leading to renal fibrosis (Yang et al., 2023). MCP-1 is a prominent chemokine produced by mononuclear/macrophage cells, finding expression in various bodily tissues including vascular endothelial cells, fibroblasts, and epithelial cells. It emerges under pro-inflammatory stimuli, mobilizing immune cells to engage in the inflammatory cascade implicated in kidney damage (He et al., 2023). The adhesion of white blood cells to vascular endothelial cells, followed by their migration through the vascular endothelium to the inflammation site, constitutes a crucial step in the inflammatory process, and adhesion molecules serve as pivotal players in this process (Singh et al., 2023). VCAM-1, a member of the immunoglobulin superfamily of adhesion molecules, serves crucially in mediating adhesion and migration of immune cells like mononuclear cells/macrophages and T lymphocytes during inflammatory responses. Its interaction facilitates the recruitment of white blood cells to inflammatory sites, amplifying the inflammatory cascade and exacerbating kidney damage (Chen Z. et al., 2023). As a widely employed inflammatory indicator in clinical settings, CRP serves as a non-antibody protein and a non-specific marker reflecting chronic low-grade inflammation. It is regularly present in the human serum, albeit in minimal amounts (1 mg/L) under normal conditions. When tissues are compromised, cytokines prompt stem cells to produce substantial quantities of CRP (Nehring et al., 2024). CRP levels can surge by over 100 times during the acute response phase, making it a distinctive marker for inflammation detection (Alsogair et al., 2023). Our findings indicate that following the establishment of the GN rat model and administration of DTX, there was a significant reduction in IL-1β, IL-18, TNF-α, TGF-β1, MCP-1, VCAM-1, and CRP in the rat serum. This suggests that DTX effectively mitigates inflammatory infiltration and lowers the levels of chemokines and adhesion factors, thereby exerting its anti-GN effects.

**FIGURE 18:**
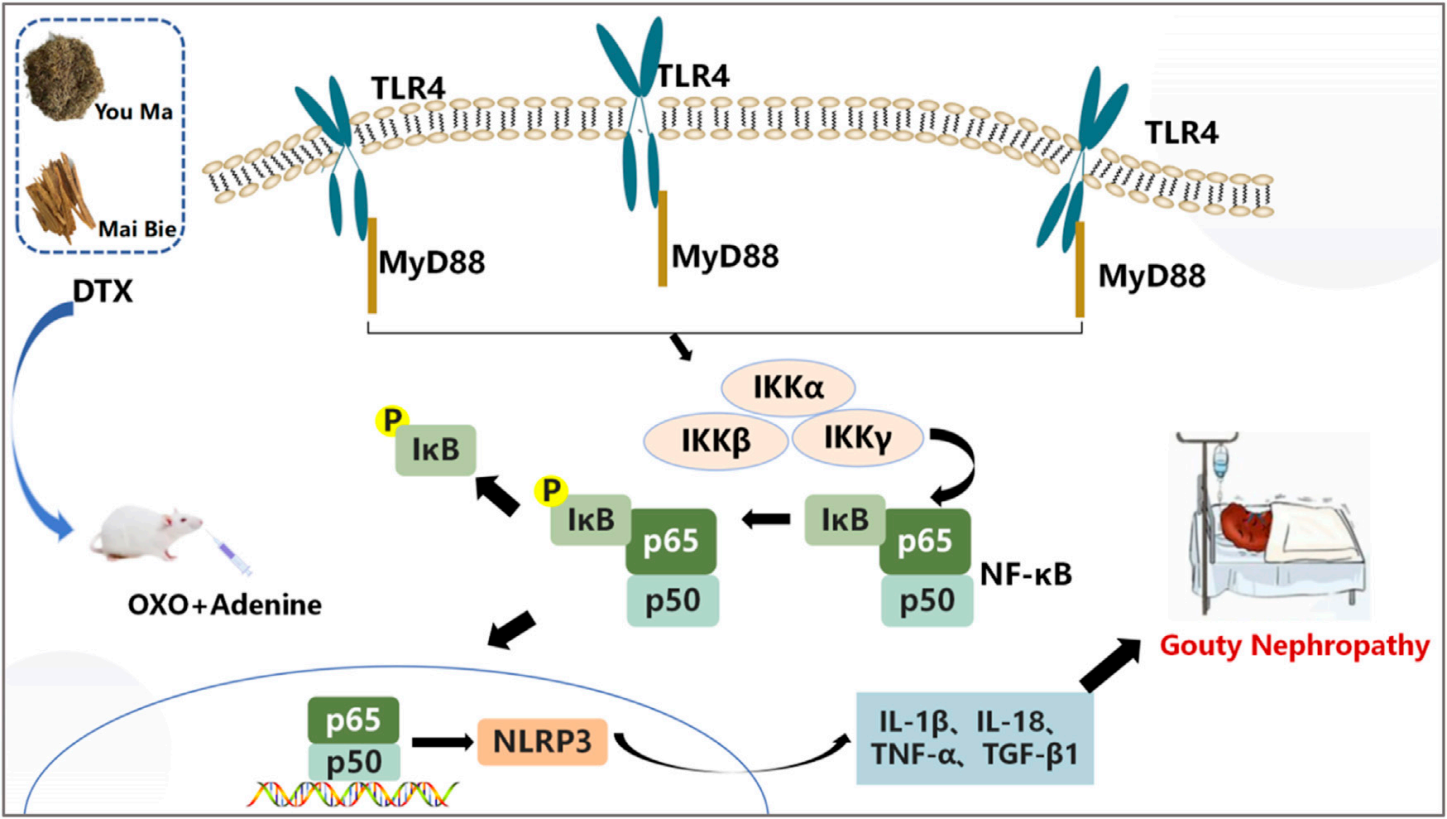
Pathogenesis of GN.

TLR signaling is intricately linked to the activation of the immune system and the pathogenesis of GN (Luo et al., 2020). TLRs serve as pivotal protein molecules bridging both specific and non-specific immune responses (Jing et al., 2015). As an important biochemical basis for GN pathogenesis, uric acid typically precipitates in the distal tubules or collecting duct lumens as insoluble urate crystals, which in turn upregulates the expression of TLR4 on renal tubule cells (Chen et al., 2024). Upon TLR4 engagement with HMGB1 secreted by immune cells, renal tubular epithelial cells are directly activated, prompting macrophage migration to the renal interstitium and initiating a cascade where TLR4 subsequently binds to MyD88. This leads to the downstream activation of NF-κB inhibitor kinase (IKKα/IKKβ/IKKγ and IKK) (Qian et al., 2024). IKK phosphorylates serine residues on IκBα, leading to the dissociation of IκBα and subsequent release of the NF-κB dimer composed of P50–P65 (YuanXiang et al., 2023). Following NF-κB translocation into the nucleus, the activation of the NLRP3 inflammasome occurs, resulting in the release of inflammatory factors and chemokines like IL-1β, TNF-β1, and MCP-1. This cascade recruits more inflammatory cells (Afonina et al., 2017), exacerbating inflammation and leading to severe kidney lesions in patients (Figure 18). Previous studies have shown that gouty nephropathy is associated with the TLR4/MyD88/NF-κB signaling pathway and that active substances can inhibit GN by modulating the TLR4/MyD88/NF-κB signaling pathway (Hao et al., 2023). Our study also revealed the activation of the TLR4/MyD88/NF-κB pathway in the GN rat model. Western blot analyses demonstrated significant upregulation of TLR4, MyD88, HMGB1, p-IKKα/IKKα, p-IKKβ/IKKβ, p-IKKγ/IKKγ, p-IκBα/IκBα, and NLRP3 expressions in renal tissues. Conversely, DTX treatment notably decreased protein expressions of TLR4, MyD88, HMGB1, p-IKKα/IKKα, p-IKKβ/IKKβ, p-IKKγ/IKKγ, p-IκBα/IκBα, and NLRP3 in renal tissues. This suggests that DTX might mitigate inflammatory factors by suppressing the TLR4/MyD88/NF-κB pathway, thereby enhancing the protective effect for the kidney. Additionally, in immunofluorescence findings, NF-κB expression in the nucleus was significantly reduced in the DTX-treated group. This could be attributed to IKK activation inhibition and reduced IκBα protein phosphorylation in the DTX-treated group, subsequently decreasing NF-κB release and inhibiting its nuclear expression in response to GN. The molecular docking results also showed that apigenin in DTX has a better affinity with TLR4, MyD88, and NF-κB. It is suggested that apigenin may directly act on the above targets, inhibiting the development of GN.

## 6 Conclusion

Our study shows the anti-GN efficacy of DTX, showcasing its ability to enhance kidney function and effectively combat kidney injury. Its mechanism of action likely involves inhibiting the inflammatory cascade by modulating the protein expression of the TLR4/MyD88/NF-κB pathway, thereby enhancing its protective effect on the kidneys. Our results reveal the previously neglected anti-GN mechanism of DTX, providing new insights and a foundation for clinical rational drug use.

## Data Availability

The raw data and image files are stored in Jianguoyun/Nutstore, available at: https://www.jianguoyun.com/p/DZj7FEEQ7_HWDBjGhckFIAA. Further queries can be directed to the corresponding authors.

## Abbreviations

α-SMA, α-smooth muscle actin; Bun, blood urea nitrogen; COL-Ⅳ, collage type Ⅳ; Cre, creatinine; CRP, C-reactive protein; CysC, cystatin C; DTX, DaiTongXiao; GN, gouty nephropathy; Hyp, hydroxyproline; IL, interleukin; MCP-1, monocyte chemoattractant protein-1; MG, microglobulin; MSU, monosodium urate; PASM, periodic Acid–Schiff–methenamine; SAA, serum amyloid A; TGF-β1, transforming growth factor-β1; TNF-α, tumor necrosis factor-α; UA, uric acid; VCAM-1, vascular cell adhesion molecule-1; XOD, xanthine oxidase.
