# Supplementary material for: DaiTongXiao improves gout nephropathy by inhibiting inflammatory response through the TLR4/MyD88/NF-κB pathway
Source: Front Pharmacol. 2024 Aug 7;15:1447241. doi: 10.3389/fphar.2024.1447241 (PMC11336418; doi:10.3389/fphar.2024.1447241)
Supplement: Supplementary file 1 [file Table1.docx]

**Table S1** Identification of the chemical composition of DTX.

| NO. | t_R_/s | ingredient | Formula | mzmed | ms2Adduct |
| --- | --- | --- | --- | --- | --- |
| 1 | 37.2 | Naphthoresorcinol | C_10_H_8_O_2_ | 161.0651 | [M+H]+ |
| 2 | 40.8 | Taurine | C_2_H_7_NO_3_S | 124.0075 | [M-H]- |
| 3 | 43.8 | Glutamine | C_5_H_10_N_2_O_3_ | 145.062 | [M-H]- |
| 4 | 44.7 | Threonine | C_4_H_9_NO_3_ | 118.0511 | [M-H]- |
| 5 | 45 | Alanine | C_3_H_7_NO_2_ | 88.0405 | [M-H]- |
| 6 | 45.1 | D-Serine | C_3_H_7_NO_3_ | 104.0354 | [M-H]- |
| 7 | 46.1 | D-Mannoheptulose | C_7_H_14_O_7_ | 209.0667 | [M-H]- |
| 8 | 46.2 | Glycine | C_2_H_5_NO_2_ | 74.0248 | [M-H]- |
| 9 | 50.3 | Quinic acid | C_7_H_12_O_6_ | 173.0457 | [M-H2O-H]- |
| 10 | 54.4 | 3-Pyridol | C_5_H_5_NO | 94.0299 | [M-H]- |
| 11 | 67.3 | Leucine | C_6_H_13_NO_2_ | 130.0875 | [M-H]- |
| 12 | 67.7 | Malic acid | C_4_H_6_O_5_ | 133.0144 | [M-H]- |
| 13 | 70.7 | Uric acid | C_5_H_4_N_4_O_3_ | 167.0212 | [M-H]- |
| 14 | 71.2 | Tyrosine | C_9_H_11_NO_3_ | 180.0668 | [M-H]- |
| 15 | 72.6 | Nicotinate | C_6_H_5_NO_2_ | 122.0248 | [M-H]- |
| 16 | 79.6 | Fumaric acid | C_4_H_4_O_4_ | 115.0038 | [M-H]- |
| 17 | 80.4 | Uridine | C_9_H_12_N_2_O_6_ | 243.0624 | [M-H]- |
| 18 | 82.7 | 3,4-Dihydroxyphenylglycol | C_8_H_10_O_4_ | 169.0508 | [M-H]- |
| 19 | 94.1 | Mesaconic acid | C_5_H_6_O_4_ | 129.0195 | [M-H]- |
| 20 | 103 | Guanosine | C_10_H_13_N_5_O_5_ | 282.0846 | [M-H]- |
| 21 | 105.3 | 2,3-Pentanedione | C_5_H_8_O_2_ | 101.0592 | [M+H]+ |
| 22 | 114.4 | 3-Hydroxymandelic acid | C_8_H_8_O_4_ | 149.0246 | [M-H2O-H]- |
| 23 | 119.3 | Angustine | C_20_H_15_N_3_O | 314.1334 | [M+H]+ |
| 24 | 121.1 | 3-Hydroxyisovaleric acid | C_5_H_10_O_3_ | 117.0558 | [M-H]- |
| 25 | 131.1 | Citraconic acid | C_5_H_6_O_4_ | 129.0195 | [M-H]- |
| 26 | 148.4 | (2R,3R)-2-(3,4-dihydroxyphenyl)-3,5,7-trihydroxy-2,3-dihydro-4H-chromen-4-one | C_15_H_12_O_7_ | 327.0463 | [M+Na]+ |
| 27 | 154.6 | (S)-Mandelic_acid_O-beta-D-Glucopyranoside | C_14_H_18_O_8_ | 315.1059 | [M+H]+ |
| 28 | 167.6 | IPRIFLAVONE | C_18_H_16_O_3_ | 281.1119 | [M+H]+ |
| 29 | 186.6 | Griffonilide | C_8_H_8_O_4_ | 167.0351 | [M-H]- |
| 30 | 197 | Ethyl 3,4-dihydroxybenzoate | C_9_H_10_O_4_ | 181.0508 | [M-H]- |
| 31 | 199.6 | Isopentenyladenine | C_10_H_13_N_5_ | 204.1222 | [M+H]+ |
| 32 | 199.7 | 2-Hydroxy-4-methoxybenzoic acid | C_8_H_8_O_4_ | 169.049 | [M+H]+ |
| 33 | 201.2 | [(2R,3S,4S,5R,6R)-6-[(2S,3S,4S,5R)-3,4-dihydroxy-2,5-bis(hydroxymethyl)oxolan-2-yl]oxy-3,4,5-trihydroxyoxan-2-yl]methyl 4-hydroxybenzoate | C_19_H_26_O_13_ | 461.1307 | [M-H]- |
| 34 | 202.6 | Tryptophan | C_11_H_12_N_2_O_2_ | 203.0827 | [M-H]- |
| 35 | 205.7 | 3,4-Dihydroxybenzoic acid (Protocatechuic acid) | C_7_H_6_O_4_ | 153.0195 | [M-H]- |
| 36 | 207 | 3,4-Dihydroxyphenylacetic acid | C_8_H_8_O_4_ | 149.0246 | [M-H2O-H]- |
| 37 | 209.6 | Acridine | C_13_H_9_N | 180.086 | [M+H]+ |
| 38 | 213.2 | 5-Hydroxyhexanoic acid | C_6_H_12_O_3_ | 131.0715 | [M-H]- |
| 39 | 213.9 | 2,3-Octanedione | C_8_H_14_O_2_ | 143.1061 | [M+H]+ |
| 40 | 214.7 | Diosbulbin B | C_19_H_20_O_6_ | 362.1583 | [M+NH4]+ |
| 41 | 220.1 | 4-Methoxyphenol | C_7_H_8_O_2_ | 125.0592 | [M+H]+ |
| 42 | 227.3 | alpha-Pinene_oxide | C_10_H_16_O | 153.1268 | [M+H]+ |
| 43 | 228.1 | (+\|-)-threo-Anethole_glycol | C_10_H_14_O_3_ | 183.1008 | [M+H]+ |
| 44 | 229.6 | Caffeic acid hexoside | C_15_H_18_O_9_ | 341.0881 | [M-H]- |
| 45 | 230.8 | 3-Hydroxy-4-methoxybenzoic acid | C_8_H_8_O_4_ | 167.0351 | [M-H]- |
| 46 | 230.9 | Phthalic acid | C_8_H_6_O_4_ | 165.0194 | [M-H]- |
| 47 | 234.3 | (E)-10-Oxo-8-decenoic_acid | C_10_H_16_O_3_ | 185.1164 | [M+H]+ |
| 48 | 235 | Protocatechualdehyde | C_7_H_6_O_3_ | 137.0245 | [M-H]- |
| 49 | 239.6 | Chlorogenic acid | C_16_H_18_O_9_ | 353.088 | [M-H]- |
| 50 | 241 | Vicenin III | C_26_H_28_O_14_ | 563.1411 | [M-H]- |
| 51 | 241.7 | (E)-3-(2-hydroxy-4-methoxy-phenyl)prop-2-enoic acid | C_10_H_10_O_4_ | 193.0508 | [M-H]- |
| 52 | 241.7 | Vanillin acetate | C_10_H_10_O_4_ | 195.0645 | [M+H]+ |
| 53 | 243.4 | 1-(.beta.-D-Glucopyranosyloxy)-7-methyl-1,4a,5,6,7,7a-hexahydrocyclopenta[c]pyran-4-carboxylic acid | C_16_H_24_O_9_ | 359.135 | [M-H]- |
| 54 | 244.1 | Picrotin_-_Picrotoxinin | C_15_H_18_O_7_ | 311.1113 | [M+H]+ |
| 55 | 245 | 4-Methylumbelliferone | C_10_H_8_O_3_ | 177.0538 | [M+H]+ |
| 56 | 249 | 7-hydroxy-2-(4-hydroxyphenyl)-8-[(2S,3R,4R,5S,6R)-3,4,5-trihydroxy-6-(hydroxymethyl)tetrahydropyran-2-yl]chromen-4-one | C_21_H_20_O_9_ | 417.1164 | [M+H]+ |
| 57 | 250.4 | Methyl (1S,4aS,7aS)-7-(hydroxymethyl)-1-[(6-O-{6-O-[(2E)-3-(4-hydroxyphenyl)-2-propenoyl]-beta-D-glucopyranosyl}-beta-D-glucopyranosyl)oxy]-1,4a,5,7a-tetrahydrocyclopenta[c]pyran-4-carboxylate | C_32_H_40_O_17_ | 697.2308 | [M+H]+ |
| 58 | 251.4 | Vicenin-1 | C_26_H_28_O_14_ | 563.1412 | [M-H]- |
| 59 | 252.5 | Homovanillic acid | C_9_H_10_O_4_ | 181.0509 | [M-H]- |
| 60 | 252.9 | Droserone | C_11_H_8_O_4_ | 205.0487 | [M+H]+ |
| 61 | 254.1 | 1-(3,4-dihydroxyphenyl)-6,7-dihydroxy-1,2-dihydronaphthalene-2,3-dicarboxylic acid | C_18_H_14_O_8_ | 357.0617 | [M-H]- |
| 62 | 254.9 | 1,4-Naphthoquinone | C_10_H_6_O_2_ | 159.0434 | [M+H]+ |
| 63 | 255.5 | Hydroxyisocaproic acid | C_6_H_12_O_3_ | 131.0715 | [M-H]- |
| 64 | 256.4 | Vanillic acid | C_8_H_8_O_4_ | 167.0352 | [M-H]- |
| 65 | 256.7 | Peperinic_acid | C_10_H_14_O_3_ | 183.1007 | [M+H]+ |
| 66 | 257.2 | (S)-(-)-Perillyl_alcohol | C_10_H_16_O | 135.1162 | [M+H-H2O]+ |
| 67 | 258 | Lonicerin | C_27_H_30_O_15_ | 593.1519 | [M-H]- |
| 68 | 258.5 | Hydroxyhydroquinone | C_6_H_6_O_3_ | 125.0245 | [M-H]- |
| 69 | 261.7 | Isoliquiritin apioside | C_26_H_30_O_13_ | 551.1735 | [M+H]+ |
| 70 | 262.5 | Neocnidilide | C_12_H_18_O_2_ | 195.137 | [M+H]+ |
| 71 | 262.8 | 2,3-Dihydroxy-1-(4-hydroxy-3-methoxyphenyl)-1-propanone | C_10_H_12_O_5_ | 213.0748 | [M+H]+ |
| 72 | 262.9 | Caffeic acid | C_9_H_8_O_4_ | 179.0351 | [M-H]- |
| 73 | 263.4 | 4-Hydroxyphenylacetaldehyde | C_8_H_8_O_2_ | 135.0452 | [M-H]- |
| 74 | 264.8 | 2-(3,4-dimethoxyphenyl)-5,6,7,8-tetramethoxy-3-[(2S,3R,4S,5S,6R)-3,4,5-trihydroxy-6-(hydroxymethyl)tetrahydropyran-2-yl]oxy-chromen-4-one | C_27_H_32_O_14_ | 581.1841 | [M+H]+ |
| 75 | 265.7 | Syringaldehyde | C_9_H_10_O_4_ | 183.0643 | [M+H]+ |
| 76 | 265.7 | Deoxyloganic acid (Not validated) | C_16_H_24_O_9_ | 359.1348 | [M-H]- |
| 77 | 266.1 | Zingerone | C_11_H_14_O_3_ | 195.1007 | [M+H]+ |
| 78 | 268.7 | Prenyletin | C_14_H_14_O_4_ | 264.1218 | [M+NH4]+ |
| 79 | 269.2 | Piperonylic acid | C_8_H_6_O_4_ | 165.0195 | [M-H]- |
| 80 | 269.7 | Prenyl_caffeate | C_14_H_16_O_4_ | 249.111 | [M+H]+ |
| 81 | 270.3 | Kanzonol C | C_25_H_28_O_4_ | 391.1977 | [M-H]- |
| 82 | 271.3 | 3-Methylcatechol | C_7_H_8_O_2_ | 125.0591 | [M+H]+ |
| 83 | 272 | alpha,alpha-Dimethylphenethyl_formate | C_11_H_14_O_2_ | 179.1058 | [M+H]+ |
| 84 | 276.1 | 5-hydroxy-2-(4-hydroxyphenyl)-7-methoxy-6-[(2S,3R,4R,5S,6R)-3,4,5-trihydroxy-6-(hydroxymethyl)tetrahydropyran-2-yl]chromen-4-one | C_22_H_22_O_10_ | 445.1148 | [M-H]- |
| 85 | 276.1 | Methyl cinnamate | C_10_H_10_O_2_ | 163.0746 | [M+H]+ |
| 86 | 276.6 | Violanthin | C_27_H_30_O_14_ | 577.1569 | [M-H]- |
| 87 | 276.9 | Leucoside | C_26_H_28_O_15_ | 581.1472 | [M+H]+ |
| 88 | 278.5 | Eriodictyol-7-neohesperidoside | C_27_H_32_O_15_ | 595.1681 | [M-H]- |
| 89 | 278.5 | Paeonilactone_A | C_10_H_14_O_4_ | 199.0956 | [M+H]+ |
| 90 | 278.7 | 3',5'-Dimethoxy-4'-hydroxyacetophenone | C_10_H_12_O_4_ | 195.0664 | [M-H]- |
| 91 | 278.8 | 5,7-Dihydroxycoumarin | C_9_H_6_O_4_ | 177.0195 | [M-H]- |
| 92 | 279.9 | Ferulate | C_10_H_10_O_4_ | 177.0538 | [M-H2O+H]+ |
| 93 | 282.1 | Samidin | C_21_H_22_O_7_ | 387.1421 | [M+H]+ |
| 94 | 282.3 | (E)-Osmundacetone | C_10_H_10_O_3_ | 179.0694 | [M+H]+ |
| 95 | 283.9 | 5-[(2S,3R,4S,5S,6R)-6-[[(2R,3R,4R)-3,4-dihydroxy-4-(hydroxymethyl)tetrahydrofuran-2-yl]oxymethyl]-3,4,5-trihydroxy-tetrahydropyran-2-yl]oxy-4-(3,4-dihydroxyphenyl)-7-hydroxy-chromen-2-one | C_26_H_28_O_15_ | 579.1364 | [M-H]- |
| 96 | 284 | Rutin | C_27_H_30_O_16_ | 611.158 | [M+H]+ |
| 97 | 285.5 | Anthraflavic_acid | C_29_H_36_O_15_ | 623.1987 | [M-H]- |
| 98 | 286.3 | Phenol | C_6_H_6_O | 93.0347 | [M-H]- |
| 99 | 287.2 | 6-Hydroxyluteolin 7-glucoside | C_21_H_20_O_12_ | 465.1007 | [M+H]+ |
| 100 | 287.9 | Iridin | C_24_H_26_O_13_ | 521.1301 | [M-H]- |
| 101 | 289.8 | Aloinoside_B | C_27_H_32_O_13_ | 565.1891 | [M+H]+ |
| 102 | 290.3 | 3-(3-Hydroxyphenyl)propanoic acid | C_9_H_10_O_3_ | 165.0559 | [M-H]- |
| 103 | 291.1 | Scutellarin | C_21_H_18_O_12_ | 461.073 | [M-H]- |
| 104 | 292.7 | Rosmarinic acid | C18H16O8 | 359.0775 | [M-H]- |
| 105 | 295.2 | 2-(3,4-dimethoxyphenyl)-3,5,7,8-tetramethoxy-chromen-4-one | C_21_H_22_O_8_ | 403.1371 | [M+H]+ |
| 106 | 295.2 | 3-(4-Methoxyphenyl)propanoic acid | C_10_H_12_O_3_ | 179.0715 | [M-H]- |
| 107 | 297.2 | Isoschaftoside | C_26_H_28_O_14_ | 563.1415 | [M-H]- |
| 108 | 301.9 | Valeric acid | C_5_H_10_O_2_ | 101.0609 | [M-H]- |
| 109 | 304.3 | 4-Hydroxycoumarin | C_9_H_6_O_3_ | 163.0382 | [M+H]+ |
| 110 | 305.7 | Luteolin 7-O-glucuronide | C_21_H_18_O_12_ | 463.0851 | [M+H]+ |
| 111 | 307.3 | Dimethyl phthalate | C_10_H_10_O_4_ | 177.0538 | [M-H2O+H]+ |
| 112 | 308.7 | (-)-Camphoric acid | C_10_H_16_O_4_ | 199.0978 | [M-H]- |
| 113 | 310 | Acetylvalerenolic_acid | C_17_H_24_O_4_ | 291.1604 | [M-H]- |
| 114 | 316.6 | Luteolin | C_15_H_10_O_6_ | 285.0405 | [M-H]- |
| 115 | 323.8 | Vulpinic acid | C_19_H_14_O_5_ | 665.1368 | [2M-2H+Na]- |
| 116 | 326.4 | Neomangiferin | C_25_H_28_O_16_ | 583.1309 | [M-H]- |
| 117 | 331 | 3-(4-Hydroxyphenyl)-1-propanol | C_9_H_12_O_2_ | 151.0767 | [M-H]- |
| 118 | 337.2 | 4',5-Dihydroxyflavone | C_15_H_10_O_4_ | 255.0641 | [M+H]+ |
| 119 | 337.3 | Pinobanksin | C_15_H_12_O_5_ | 253.0508 | [M-H2O-H]- |
| 120 | 337.5 | Isosinensetin | C_20_H_20_O_7_ | 373.1267 | [M+H]+ |
| 121 | 342.7 | Royal jelly acid | C_10_H_18_O_3_ | 185.1185 | [M-H]- |
| 122 | 344.4 | Quercetin | C_15_H_10_O_7_ | 301.0357 | [M-H]- |
| 123 | 344.5 | Acetylpanaxydol | C_19_H_26_O_3_ | 303.194 | [M+H]+ |
| 124 | 346 | Semilicoisoflavone B | C_20_H_16_O_6_ | 353.1004 | [M+H]+ |
| 125 | 348.8 | N-Methyltyramine | C_9_H_13_NO | 152.1063 | [M+H]+ |
| 126 | 350.1 | Capsiate | C_18_H_26_O_4_ | 307.1891 | [M+H]+ |
| 127 | 355.8 | Anwuligan | C_20_H_24_O_4_ | 329.1733 | [M+H]+ |
| 128 | 359.7 | Demethoxycapillarisin | C_15_H_10_O_6_ | 287.0537 | [M+H]+ |
| 129 | 360.2 | Brefeldin A | C_16_H_24_O_4_ | 279.1604 | [M-H]- |
| 130 | 364.6 | Xanthumin | C_17_H_22_O_5_ | 307.1527 | [M+H]+ |
| 131 | 366.7 | Falcarinol | C_17_H_24_O | 245.1887 | [M+H]+ |
| 132 | 367.3 | [(3aS,4S,5S,6E,10E,11aR)-6-formyl-5-methoxy-10-methyl-3-methylidene-2-oxo-3a,4,5,8,9,11a-hexahydrocyclodeca[b]furan-4-yl] 2-methylpropanoate | C_20_H_26_O_6_ | 380.2048 | [M+NH4]+ |
| 133 | 368 | Apigenin | C_15_H_10_O_5_ | 269.0458 | [M-H]- |
| 134 | 368.5 | Kaempferol | C_15_H_10_O_6_ | 285.0407 | [M-H]- |
| 135 | 368.7 | [8]-Dehydroshogaol | C_19_H_26_O_3_ | 303.1941 | [M+H]+ |
| 136 | 370 | alpha-Amylcinnamyl_acetate | C_16_H_22_O_2_ | 247.1681 | [M+H]+ |
| 137 | 375.1 | 8-acetyl-7-hydroxy-chromen-2-one | C_11_H_8_O_4_ | 203.0352 | [M-H]- |
| 138 | 378.8 | (2E)-3-[(1R,4S,7R,7aR)-1-Hydroxy-3,7-dimethyl-2,4,5,6,7,7a-hexahydro-1H-inden-4-yl]-2-methylacrylic acid | C_15_H_22_O_3_ | 273.1473 | [M+Na]+ |
| 139 | 379.2 | Spegatrine | C_20_H_25_N_2_O_2_ | 326.194 | [M+H]+ |
| 140 | 379.3 | 2'-Hydroxy-5'-methylacetophenone | C_9_H_10_O_2_ | 149.061 | [M-H]- |
| 141 | 379.4 | (3aS,4S,7aR)-4-Hydroxy-5-[(2S)-5-hydroxy-2-pentanyl]-6-methyl-3-methylene-3a,4,7,7a-tetrahydro-1-benzofuran-2(3H)-one | C_15_H_22_O_4_ | 289.1421 | [M+Na]+ |
| 142 | 380.5 | Periplocymarin | C_30_H_46_O_8_ | 533.3125 | [M-H]- |
| 143 | 383.1 | Ginsenoyne_E | C_17_H_22_O_2_ | 259.168 | [M+H]+ |
| 144 | 386.8 | Ponicidin/Rubescensin B | C_20_H_26_O_6_ | 363.1786 | [M+H]+ |
| 145 | 391.4 | Sparteine | C_15_H_26_N_2_ | 235.2045 | [M+H]+ |
| 146 | 391.9 | Capsaicin | C_18_H_27_NO_3_ | 328.1892 | [M+Na]+ |
| 147 | 392.8 | Propyl paraben | C_10_H_12_O_3_ | 179.0715 | [M-H]- |
| 148 | 395.8 | Dodecanedioic acid | C_12_H_22_O_4_ | 229.1446 | [M-H]- |
| 149 | 399.9 | angoletin | C_18_H_20_O_4_ | 299.1316 | [M-H]- |
| 150 | 400.2 | Glycocholic acid | C_33_H_37_N_5_O_5_ | 464.3021 | [M-H]- |
| 151 | 401.9 | 5,7-dihydroxy-2-phenyl-chroman-4-one | C_15_H_12_O_4_ | 255.0664 | [M-H]- |
| 152 | 402.6 | 8-Gingerol | C_19_H_30_O_4_ | 303.1968 | [M-H2O-H]- |
| 153 | 403.3 | Eudesmin | C_22_H_26_O_6_ | 387.1782 | [M+H]+ |
| 154 | 408.5 | (8)-Gingerol | C_19_H_30_O_4_ | 323.2202 | [M+H]+ |
| 155 | 412.5 | Cembratetraene-16,2:19,6-diolide | C_20_H_24_O_4_ | 329.1733 | [M+H]+ |
| 156 | 415.1 | Arnebinol | C_16_H_20_O_2_ | 245.1524 | [M+H]+ |
| 157 | 417.3 | 6,7-Dihydrotabersonine | C_21_H_26_N_2_O_2_ | 339.215 | [M+H]+ |
| 158 | 432.8 | testolactone | C_19_H_24_O_3_ | 301.1785 | [M+H]+ |
| 159 | 433.8 | Pisiferal | C_20_H_28_O_2_ | 301.215 | [M+H]+ |
| 160 | 437.7 | Eucalyptin | C_19_H_18_O_5_ | 327.1213 | [M+H]+ |
| 161 | 439.6 | 10-Acetylpanaxytriol | C_19_H_28_O_4_ | 321.2045 | [M+H]+ |
| 162 | 448.5 | Euscaphic acid | C_30_H_48_O_5_ | 487.3435 | [M-H]- |
| 163 | 449.9 | Pelargonic acid | C_9_H_18_O_2_ | 157.1236 | [M-H]- |
| 164 | 458.4 | 2-[(1R,6R)-6-isopropenyl-3-methyl-cyclohex-2-en-1-yl]-5-propyl-benzene-1,3-diol | C_19_H_26_O_2_ | 287.1991 | [M+H]+ |
| 165 | 461.5 | Ginsenoyne_F | C_19_H_24_O_3_ | 301.1785 | [M+H]+ |
| 166 | 463.2 | 9-hydroxy-7-isopropyl-1,4a-dimethyl-2,3,4,9,10,10a-hexahydrophenanthrene-1-carboxylic acid | C_20_H_28_O_3_ | 315.1968 | [M-H]- |
| 167 | 468.2 | 10-Undecenoic acid | C_11_H_20_O_2_ | 183.1392 | [M-H]- |
| 168 | 469.8 | ethyl octanoate | C_10_H_20_O_2_ | 171.1392 | [M-H]- |
| 169 | 476 | Cafestol | C_20_H_28_O_3_ | 339.1913 | [M+Na]+ |
| 170 | 476.4 | (+)-Usnic acid | C_18_H_16_O_7_ | 343.0823 | [M-H]- |
| 171 | 476.5 | (1R,2R,4aS,6aS,6bR,10S,12aR)-1,10-dihydroxy-1,2,6a,6b,9,9,12a-heptamethyl-2,3,4,5,6,6a,7,8,8a,10,11,12,13,14b-tetradecahydropicene-4a-carboxylic acid | C_30_H_48_O_4_ | 471.3485 | [M-H]- |
| 172 | 477.8 | 16-Hydroxypalmitic acid | C_16_H_32_O_3_ | 271.228 | [M-H]- |
| 173 | 489.7 | (2S,4aS,6aS,6bR,10S,12aS,14bS)-10-hydroxy-2,4a,6a,6b,9,9,12a-heptamethyl-13-oxo-3,4,5,6,6a,7,8,8a,10,11,12,14b-dodecahydro-1H-picene-2-carboxylic acid | C_30_H_46_O_4_ | 469.3328 | [M-H]- |
| 174 | 490 | Undecanoic acid | C_11_H_22_O_2_ | 185.1548 | [M-H]- |
| 175 | 508.2 | Dodecanoic acid | C_12_H_24_O_2_ | 199.1704 | [M-H]- |
| 176 | 514.8 | Dehydroabietic_acid | C_20_H_28_O_2_ | 299.2017 | [M-H]- |
| 177 | 524.1 | Eicosapentaenoic acid | C_20_H_30_O_2_ | 301.2174 | [M-H]- |
| 178 | 530.4 | alpha-Linolenic acid | C_18_H_30_O_2_ | 277.2173 | [M-H]- |
| 179 | 538.8 | Continentalic acid | C_20_H_30_O_2_ | 301.2174 | [M-H]- |
| 180 | 539.3 | Myristic acid | C_14_H_28_O_2_ | 227.2016 | [M-H]- |
| 181 | 542.1 | (E)-5-(1,2,4a,5-tetramethyl-2,3,4,7,8,8a-hexahydronaphthalen-1-yl)-3-methyl-pent-2-enoic acid | C_20_H_32_O_2_ | 303.2329 | [M-H]- |
| 182 | 543.4 | cis-9-Palmitoleic acid | C_16_H_30_O_2_ | 253.2173 | [M-H]- |
| 183 | 547.7 | Linoleic acid | C_18_H_32_O_2_ | 279.2328 | [M-H]- |
| 184 | 554 | cis-8,11,14-Eicosatrienoic acid | C_20_H_34_O_2_ | 305.2487 | [M-H]- |
| 185 | 566 | Oleic acid | C_18_H_34_O_2_ | 281.2485 | [M-H]- |
| 186 | 568.8 | cis-11.14-Eicosadienoic acid | C_20_H_36_O_2_ | 307.2643 | [M-H]- |
| 187 | 585 | trans-11-Eicosenoic acid | C_20_H_38_O_2_ | 309.2799 | [M-H]- |
| 188 | 595.2 | Nonadecanoic acid | C_19_H_38_O_2_ | 297.2799 | [M-H]- |
| 189 | 624.5 | Nervonic acid | C_24_H_46_O_2_ | 365.3424 | [M-H]- |
